# Analysis and validation of silica-immobilised BST polymerase in loop-mediated isothermal amplification (LAMP) for malaria diagnosis

**DOI:** 10.1007/s00216-022-04131-2

**Published:** 2022-06-03

**Authors:** Dushanth Seevaratnam, Felix Ansah, Yaw Aniweh, Gordon A. Awandare, Elizabeth A. H. Hall

**Affiliations:** 1 Department of Chemical Engineering and Biotechnology, University of Cambridge, Philippa Fawcett Drive, Cambridge, CB3 0AS UK; 2 West African Centre for Cell Biology of Infectious Pathogens, University of Ghana, P. O. Box LG 54, Legon-Accra, Ghana

**Keywords:** LAMP, Malaria, BST polymerase, Local production, Low cost

## Abstract

*Bacillus stearothermophilus* large fragment (BST_LF_) DNA polymerase is reported, isolated on silica via a fused R5 silica-affinity peptide and used in nucleic acid diagnostics. mCherry (mCh), included in the fusion construct, was shown as an efficient fluorescent label to follow the workflow from gene to diagnostic. The R5 immobilisation on silica from cell lysate was consistent with cooperative R5-specific binding of R5_2_-mCh-FL-BST_LF_ or R5_2_-mCh-H10-BST_LF_ fusion proteins followed by non-specific protein binding (including *E. coli* native proteins). Higher R5-binding could be achieved in the presence of phosphate, but phosphate residue reduced loop-mediated isothermal amplification (LAMP) performance, possibly blocking sites on the BST_LF_ for binding of β- and γ-phosphates of the dNTPs. Quantitative assessment showed that cations (Mg^2+^ and Mn^2+^) that complex the PPi product optimised enzyme activity. In malaria testing, the limit of detection depended on *Plasmodium* species and primer set. For example, 1000 copies of *P. knowlesi* 18S rRNA could be detected with the P.KNO-LAU primer set with Si-R5_2_-mCh-FL-BST_LF_ , but 10 copies of *P. ovale* 18S rRNA could be detected with the P.OVA-HAN primer set using the same enzyme. The Si-immobilised BST_LF_ outperformed the commercial enzyme for four of the nine *Plasmodium* LAMP primer sets tested. Si-R5_2_-mCh-FL-BST_LF_ production was transferred from Cambridge to Accra and set up de novo for a trial with clinical samples. Different detection limits were found, targeting the mitochondrial DNA or the 18S rRNA gene for *P. falciparum.* The results are discussed in comparison with qPCR and sampling protocol and show that the Si-BST_LF_ polymerase can be optimised to meet the WHO recommended guidelines.

**Graphical abstract:**

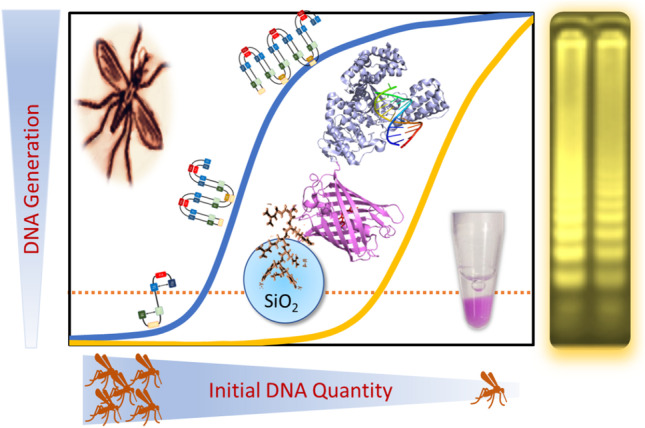

**Supplementary Information:**

The online version contains supplementary material available at 10.1007/s00216-022-04131-2.

## Introduction

Malaria has been a major concern for over 30 years in Africa. It is a life-threatening disease that is typically caused by the transmission of a *Plasmodium* parasite from an infected mosquito’s bite with symptoms of high fever, headaches and nausea [1]. Of the various species of *Plasmodium*, there are 5 that are usually cited as infectious towards humans; they are *Plasmodium falciparum* (*P. falciparum*), *P. vivax*, *P. malariae*, *P. ovale* and *P. knowlesi*. The WHO reported that in 2020 there were 241 million cases of malaria and 627,000 deaths due to the infection (over 90% occur in Africa), an increase from previous years due to the impact on the effected regions by the COVID-19 pandemic [2]. This poses a high burden on the economy of poor countries and it has been suggested that countries with malaria endemics have 1.3% lower economic growth rates relative to economically comparable malaria-free countries [3]. Early and accurate diagnosis of malaria is essential for life-saving treatment and effective disease management as well as to provide malaria surveillance.

In parts of rural Africa, malaria is often diagnosed based on clinical symptoms, such as fever, without use of diagnostics. Where diagnostics are used, microscopy remains the cheapest option, at ~ $0.26/test slide, but the accuracy of the diagnosis depends on the equipment, reagents and the skills of the personnel. A 2018 study in Equatorial Guinea showed that ~ 20% of “negative” samples detected by microscopy were false negatives when compared with the gold standard PCR test [4]. A number of rapid diagnostic tests (RDTs) detecting the presence of parasite antigens in the patient’s blood have also been available for the last decade or so, but here too ~ 13% of negative samples detected were false negatives based on PCR [4] and the test is more expensive to use. In addition to the consequences of delayed treatment due to false negatives, unreliable results lead to an overall low trust by clinicians in these tests. As a result, anti-malarial drugs are often prescribed even when microscopy or RDT tests are negative, and in some cases, the RDTs have also produced false positives when the patient has a fever due to another disease. Taken together, this has another impact: in parts of Africa, up to 60% of patients prescribed anti-malaria treatment actually suffer from another disease [5].

However, the gold standard PCR is not suitable for testing in remote rural communities with no laboratory facilities and, in general, is also too expensive to provide a viable solution [6]. Many African countries are only able to spend $8 to $129 per capita on health, compared with high-income countries that spend above $4000 [7], so while there is a need for PCR diagnostics, there is a fiscal and facility barrier to its use.

In terms of other nucleic acid tests, loop-mediated isothermal amplification (LAMP) has been shown as a promising alternative to PCR [8, 9] and has been reported to be an effective diagnostic assay for a variety of infectious diseases [10, 11]. LAMP-based DNA amplification has been reported to be one of the most efficient isothermal amplification techniques, capable of competing with PCR and not requiring such expensive thermocycle control [12, 13]. LAMP achieves this by using four to six primers that target six to eight sites, along a ~ 250 bp long sequence of DNA, and a polymerase that is capable of separating dsDNA without temperature denaturation (strand displacement) [14]. However, while this isothermal system has potential for easier configuration for use out of the laboratory in low resource areas, an expensive polymerase enzyme is still required. Seventy to eighty percent of the material cost for a point-of-care diagnostic can be attributed to the high cost of the enzymes [15]. In fact, the financial burden caused by the cost of supply of enzymes and other affinity proteins can also limit their use in diagnostics that are restricted to laboratory environments and expensive equipment like PCR. Typically, these materials are produced in higher-income countries without global purchasing price parity.

With the growth of “garage biotech” enabling synthetic biology techniques to be applied without such major investment in laboratory facilities, solutions could be driven by empowering small enterprises with the ability to develop a more locally distributed manufacturing model, to produce enzymes and bring social innovation forward [16, 17]. Taking this approach, in combination with protein engineering for improved functionality, ease of use and cheaper appropriate purification of these enzyme materials for use in diagnostics, may help bring these essential analytical reagents to low- and middle-income countries (LMICs) in a sustainable manner.

We have previously reported the use of protein engineering to produce a fusion construct containing the R5 silaffin-tag with a red fluorescent mCherry protein together with a functional enzyme—sarcosine oxidase [15, 18, 19]. The latter is the bioanalytical reagent for sarcosine determination in urine. The positively charged R5 silaffin-tag enables the use of negatively charged silica particles (through protein immobilisation) to concentrate the targeted enzyme from other native *E. coli* proteins and DNA, and to use particle precipitation and rapid buffer exchange, without the need for filters and/or dialysis to isolate and use an enzyme, still attached to the silica, that is fit for use in a diagnostic device. Meanwhile, fluorescence measurements of the fused mCherry can be utilised as a label to follow the workflow, providing reliable insight into the status of protein production and immobilisation, as well as indicating the quality of stored enzyme.

Building on this previous work on a recombinant protein architecture for facilitated production of oxidoreductase classed enzymes for diagnostics [15], we examine whether the protein architecture can be adapted and used for large fragment *Geobacillus stearothermophilus* (BST) DNA polymerase (BST_LF_), a transferase classed enzyme and protein of choice for LAMP-based DNA amplification. A route to distributed low-cost production of polymerases could widen their availability and use for malaria testing as well as nucleic acid testing for other febrile diseases. The goal of this work was to evaluate the performance of BST_LF_, when produced by recombinant techniques as a fusion protein, with protein labelling and silica-affinity immobilisation, exploring its application in clinical identification of *P. falciparum* infection at the West African Centre for Cell Biology of Infectious Pathogens, WACCBIP, a leading institution in molecular and cellular studies of malaria in Ghana.

## Materials and methods

Lysozyme from chicken egg white, potassium chloride (KCl), tris(hydroxymethyl)aminomethane (Tris), ethylenediaminetetraacetic acid (EDTA), dithiothreitol (DTT), Triton X-100, manganese chloride (MnCl_2_), sodium hydroxide (NaOH), hydrochloric acid (HCl), Coomassie Brilliant Blue, N,N,N’,N’-tetramethyl-ethylenediamine (TEMED), sodium dodecyl sulfate (SDS), acrylamide/Bis-acrylamide, ammonium persulfate (APS), glycine, bromophenol blue, agarose and acetic acid were obtained from materials sourced from Sigma-Aldrich. LB agar, LB broth, kanamycin, ampicillin and isopropyl β-D-1-thiogalactopyranoside (IPTG) were obtained from Melford Laboratories. Q5 high-fidelity DNA polymerase, Klenow fragment, BST DNA Polymerase Large Fragment, NEBuffer™ 2, isothermal amplification buffer II, ThermoPol® reaction buffer, magnesium sulfate (MgSO_4_), deoxynucleotide solution mix, BbsI-HF®, MfeI-HF®, rCutSmart™ buffer, 6 × DNA loading dye, nuclease-free water, NEB® Turbo Competent *E. coli* (High Efficiency) and BL21 (DE3) competent *E. coli* were obtained from New England Biolabs. SYBR™ safe DNA gel stain, SYBR™ Green I Nucleic Acid Gel Stain was obtained from Thermo Fisher Scientific. Silica gel 60 (< 63 µm) was obtained from Fluka. QIAquick gel extraction kit, QIAprep Spin Miniprep Kit and QIAamp® DNA Mini Kit were obtained from QIAGEN. Ni–NTA His·Bind® Resins were obtained from Novagen.

### Protein design of R5_2_-mCh-H10-BST_LF_, R5_2_-mCh-FL-BST_LF_ (Fig. 1)

The BST_LF_ sequence was ordered from GeneArt™ Gene Synthesis. The gene was cloned into a R5_2_-mCh-monomeric sarcosine oxidase (mSOx)-R5 pET24a( +) construct previously designed [15] and prepared in-house using Klenow assembly. PCR (method outlined below) using the primers from Table S1 was used to generate the amplicons for R5_2_-mCh-histidine × 10 (H10)-BST_LF_ and R5_2_-mCh-Flexible Linker (FL)-BST_LF_. Klenow assembly (method outlined below) was performed using the following gel-purified amplicon sets:

1. pET24a( +) – 1, pET24a( +) – mCh-H10 and H6-BST_LF_
2. pET24a( +) – 1, pET24a( +) – mCh-FL and FL-BST_LF_

Assembled products were transformed into NEB Turbo cells (manufacturer heat shock protocol), cultured, miniprepped (manufacturer’s protocol) and validated through DNA sequencing services provided by the Department of Biochemistry, University of Cambridge (sequences given in Supplementary Table S2). Successfully sequenced plasmids were transformed into BL21 (DE3) cells (manufacturer heat shock protocol) for protein expression. Viable colonies were stored in 25% glycerol stocks at − 80 °C.

### PCR protocol for Klenow amplicons

The 50µL Q5 high-fidelity PCR reactions contained 1 × Q5 reaction buffer, dNTP (0.2 mM each), forward and reverse primers (0.5 µM each), 1–10 ng of template DNA and 0.01U of Q5 high-fidelity DNA polymerase. The temperature profile for the 30 cycle PCR is listed in Table S3.

### Klenow assembly

Primers (Table S1) were designed to generate amplicons with overlapping ends (15–20 bp). The resulting DNA fragments (30 ng each) were incubated with 5U of DNA polymerase I, Large (Klenow) Fragment and 1 × NEBuffer™ 2 at 37 °C for 30 min.

### Expression of R5_2_-mCh-H10-BST_LF_ and R5_2_-mCh-FL-BST_LF_

Both the BST_LF_ pET24a( +) plasmids were expressed in BL21 (DE3) cells. Twenty milliliters of LB broth overnight cultures was grown with 50 µg/mL kanamycin at 37 °C, 225 rpm. The next morning, the overnight culture was used to inoculate 200 mL of fresh LB broth with 50 µg/mL kanamycin, and incubated at 37 °C, 225 rpm until an OD_600_ of 0.6–0.8 was reached. Protein expression was then induced with 1 mM IPTG and incubated at 37 °C, 225 rpm for 4.5 to 6 h. After induction, the culture was aliquoted into 50 mL centrifuge tubes and centrifuged at 3750 rpm, 4 °C for 30 min. Samples were decanted and the pellets were stored at − 20 °C.

### Protein purification using nickel His-Bind Resin

Expressed *E. coli* pellets were thawed and suspended in 5 mL of 1 mg/mL lysozyme in H_2_O and incubated at room temperature for 30 min. The lysate was then centrifuged at 13krpm for 30 min as 1 mL aliquots (Biofuge pico, Heraeus Instruments). Nickel His-Bind Resin columns were prepared using the manufacturer’s protocol. The columns were first washed with a 5 × bed volume of buffer A (20 mM sodium phosphate, 500 mM sodium chloride, 20 mM imidazole, pH 7.8) before loading the supernatant of the lysed cell pellet onto the resin bed and incubating the columns at room temperature for 30 min. The bound cell suspension was washed with a 3 × bed volume of buffer A followed by a 5 × bed volume of washing buffer (20 mM sodium phosphate, 500 mM sodium chloride, 20 mM imidazole, 50 mM imidazole, pH 7.8). The protein was then eluted with a 2 × bed volume of elution buffer (20 mM sodium phosphate, 500 mM sodium chloride, 20 mM imidazole, 250 mM imidazole, pH 7.8). The eluted protein was desalted, buffer exchanged into BST storage buffer (2.5 mM Tris–HCl, 30 mM KCl, 1 mM DTT, 25 µM EDTA, 0.1% Triton X-100, pH 7.2), concentrated using Amicon® Ultra 0.5 mL centrifugal filters (Merck Millipore Ltd) and stored in 4 °C. Finally, protein concentrations were quantified using a Bradford assay (Bio-Rad) by following the manufacturer’s protocol.

### Protein purification using silica immobilisation

Expressed *E. coli* pellets were thawed and suspended in 5 mL of 1 mg/mL lysozyme in BST storage buffer or phosphate-buffered saline (PBS) and incubated at room temperature for 30 min. The lysate was then centrifuged at 13krpm for 30 min as 1 mL aliquots. Silica particles were suspended at a concentration of 20 mg/mL with BST storage buffer or PBS and incubated at room temperature for at least 30 min. The silica particles were aliquoted based on desired silica mass and centrifuged for 5 min at 13krpm, and decanted. The supernatant of the cell lysate was then added directly to the silica and incubated at room temperature for 30 min with a vortex (VariMix, SciEquip) every 10 min. The protein absorbed silica was centrifuged for 5 min at 13krpm, and the supernatant/flow-through was collected to measure immobilisation efficiency through fluorescence intensity and SDS-PAGE analysis. The percentage of immobilised recombinant protein was calculated by measuring the difference in fluorescence intensity of supernatant mCherry (ex/em 580 nm/610 nm) before and after immobilisation (Cary Eclipse Fluorescence Spectrophotometer). The silica-immobilised protein was washed with 20 × final suspension volume of BST storage buffer or PBS, suspended at a ratio of 5µL of buffer per 1 mg of silica and stored at 4 °C.

### SDS-PAGE analysis of recombinant proteins

8% SDS-PAGE gels were used to study R5_2_-mCh-H10-BST_LF_ and R5_2_-mCh-FL-BST_LF_. Samples (including silica with absorbed protein) were prepared with 1 × loading dye (62.5 mM Tris–HCl pH 6.8, 2.5% SDS, 0.002% Bromophenol Blue, 0.7135 M β-mercaptoethanol, 10% glycerol), heated to 95 °C for 5 min and then centrifuged at 13krpm, 4 °C for 5 min. Nine microliters of the denatured sample was loaded onto the gel and electrophoresed for 15 min at 100 V and 45 min at 175 V. After electrophoresis, the gel is stained for 2 h with Quick Stain Coomassie (Generon) and then destained with water overnight. The SDS-PAGE gels were imaged using Syngene G:Box using the following GeneSys image acquisition software settings: focus at 170, iris at 8.3, zoom at 271 and selected the gel as the exposure area. The displayed image is then analysed using ImageJ. First, the image undergoes a black/white inversion, and the intensity of the band is measured through ImageJ’s Integrated Density function. Band intensity to protein quantity is determined through a BSA standard ranging from 0.23 to 1.5 µg.

### Generating target DNA for malaria assays

The *P. vivax* 18S rRNA (GenBank accession no. U03079.1), *P. ovale* 18S rRNA (GenBank accession no. L48986.1) and *P. knowlesi* 18S rRNA (GenBank accession no. L07560.1) template DNA was purchased from Twist Bioscience. The *P. falciparum* 18S rRNA (GenBank accession no. AF145334.1) and *P. malariae* 18S rRNA (GenBank accession no. AF145336.1) template DNA were received from the West African Centre for Cell Biology of Infectious Pathogens (WACCBIP), University of Ghana. The plasmids were transformed into NEB Turbo cells using the manufacturer’s recommended heat shock protocol. *E. coli* containing the plasmid were grown in 5 mL LB broth overnight cultures with 50 µg/mL of kanamycin (37 °C, 225 rpm). The next day, the bacteria were retrieved via centrifugation (3750 rpm for 30 min), and the template DNA was extracted using a QIAprep® Spin Miniprep Kit. DNA concentrations were determined using a NanoDrop ND-1000 Spectrophotometer. Genomic DNA from lab-cultured parasite strain *P. falciparum* Dd2 and clinical venous blood samples were extracted using a QIAamp® DNA Mini Kit. The final elution volume varied between kit users from the WACCBIP team, ranging from 50µL (Cape Coast samples) to 400µL (Baiden-Ghartey Hospital), as some clinical trial workers prioritised DNA concentration while others focused on total DNA yield. In addition, eluted venous blood samples were visually screened for haemoglobin contamination. Samples that were visually yellow (Figure S1) were not included in the data set since blood contamination would interfere with the comparative PCR assay. Genomic DNA concentrations of Dd2 were determined using a NanoDrop One (Thermo Scientific), diluted with nuclease-free water accordingly and stored at − 20 °C.

### Primers for malaria LAMP assay

To test the LAMP assay using the fast production R5_2_-mCh-H10-BST_LF_ and R5_2_-mCh-FL-BST_LF_ for malaria detection, 8 primer sets, shown in Table S4 and Table S5, were obtained from the literature targeting the 18S rRNA gene (2 for *P. falciparum*, 2 for *P. vivax*, 2 for *P. ovale*, 1 for *P. malariae* and 1 for *P. knowlesi*) and 1 set targeting the mitochondrial DNA of *P. falciparum*. These primer sets are referred to in text as P.FAL-LAU, P.MAL-LAU, P.VIV-LAU, P.OVA-LAU, P.KNO-LAU, P.FAL-HAN, P.VIV-HAN, P.OVA-HAN and P.FAL-MITO in reference to the target and the author reporting the primer set.

### BST_LF_ activity assay

BST_LF_ activity was measured using a 2-step protocol. The first step was to perform a 2-primer strand displacement isothermal amplification. The 25µL reaction was comprised of 1 × ThermoPol® reaction buffer, 6 mM of MgSO_4_, 0.4 mM of each dNTP, 0.4 µM of the F3 and B3 primers from the P.KNO-LAU set, 100 copies of *P. knowlesi* 18S rRNA encoded plasmid and the BST_LF_ being tested. The reaction was performed at 65 °C for 15 min followed by a 5-min inactivation step at 80 °C. The second step was to use qPCR to quantify the number of copies of the target gene generated within the 15-min amplification period. This was done by adding 3µL of the strand displacement isothermal amplification sample to a 25µL qPCR reaction comprised of 1 × Q5 reaction buffer, 200 µM dNTP, 0.5 µM of the F3 and B3 primers from the P.KNO-LAU set, 0.005U of Q5 High-Fidelity DNA Polymerase and 1 × SYBR green. Using an ABI PRISM® 7000 Sequence Detection System thermocycler, a 50 cycle PCR was run using the conditions in Table S6. The fluorescence intensity of the samples was analysed using the ABI PRISM® 7000 SDS Software. In principle, BST_LF_ with higher enzymatic activity would generate more DNA copies in the first step, resulting in a lower cycle threshold in the second step.

### LAMP assay

The 25µL LAMP reaction mix contained 1.6 µM of FIP and BIP, 0.2 µM of F3 and B3, 0.4 µM of LPF and LPB, 1.4 mM of each dNTP, 6 mM of MgSO_4_ and 1µL of template DNA, 2µL of clinical DNA or H_2_O for negative (-ve) controls. For the commercial LAMP assay, the reaction would also contain 8U of commercial BST_LF_ and 1 × ThermoPol® reaction buffer. The silica-immobilised LAMP assays utilised 0.4 mg of protein-immobilised silica with 0.2 × isothermal buffer II and 1 mM MnCl_2_. The reactions were incubated at 65 °C for 15–90 min followed by a 5-min inactivation step at 80 °C.

### Restriction digest assay

Both the P.FAL-LAU and P.OVA-LAU LAMP products were analysed using BbsI-HF®, and MfeI-HF® was used to analyse the P.VIV-HAN and P.OVA-HAN LAMP products. Each 50µL reaction was comprised of 1 × rCutSmart™ buffer, 20U of restriction enzyme and 5µL of LAMP product. The samples were incubated at 37 °C for 16 h. Analysis was conducted via 2.5% agarose gels and 20µL of digested product compared against 2µL of undigested LAMP product.

### Diagnostic PCR assay and LAMP comparison

Ethical approval was obtained to test blood samples obtained from malaria patients who were undergoing a trial on artemisinin-based combination therapy (“The Effects of Artemisinin-Based Combination Therapy (Act) on The Dynamics of *Plasmodium falciparum*, *P. Malariae* and *P. ovale* Infections in Ghana” (GHS-ERC:005/12/17)). Patient consent was obtained and the samples were anonymised providing only the regional testing location and the outcome of a PCR test performed in 20µL reaction volumes to process the clinical samples. These results provided a ‘gold standard’ reference for the LAMP comparison on the same samples. The LAMP assay followed the protocol above. Initial testing received approval from the Human Biology Research Ethics Committee (HBREC) Cambridge, approval HBREC.2019.10: (“Specific, Sensitive and Rapid Detection of *Plasmodium* Infection in Malaria Patients and mosquitoes by Loop-mediated Isothermal Amplification (LAMP) and Recombinase Polymerase Amplification (RPA) assays”). The PCR reaction contained 1 × OneTaq® hot start master mix (New England BioLabs Inc.), 0.25 µM of forward (5’- TTAAACTGGTTTGGGAAAACCAAATATATT) and reverse (5’-CCTGTTGTTGCCTTAAACTTC) primers [20] and 1µL of clinical DNA. The temperature conditions for the 50 cycle PCR are listed in Table S7.

### Quantitative PCR assay

The qPCR was performed in 15µL reactions containing 1 × Luna® Universal qPCR Master Mix (New England BioLabs Inc.), 0.2 µM of forward (5’ – AAGTAGCAGGTCATCGTGGTT) and reverse (5’ – TTCGGCACATTCTTCCATAA) primers [21] and 2µL of clinical DNA. Using a QuantStudio™ 5 Real-Time PCR System (Thermo Fisher Scientific), the 40-cycle qPCR assay was run using the conditions listed in Table S8. The melt curves were analysed using the QuantStudio™ Design and Analysis Software.

### Agarose gel electrophoresis

The nucleic acid amplification products were verified by agarose gel electrophoresis. One to two percent (w/v) of agarose gels were prepared using agarose dissolved in TAE buffer (40 mM Tris, 20 mM acetic acid, 1 mM EDTA, pH 8.0) and stained with 1 × SYBR™ safe DNA gel stain. Five microliters of LAMP products was mixed with 1µL of 6 × DNA loading dye and electrophoresed for 50 min at 85 V. The agarose gels were imaged using Syngene G:Box.

## Results and discussion

### Approach to recombinant protein design for nucleic acid polymerase

Based on cost and availability barriers to the use of diagnostics in low resourced countries, the focus of this work was to explore the wider application of a protein architecture developed previously by Hall et al. [15] to the production of a nucleic acid polymerase. The architecture has the potential to provide a simpler and cost-effective workflow from gene to working diagnostic, in a format that could be easily transferred into the production of long fragment BST (BST_LF_) DNA polymerase at new (potentially more remote) sites. This enzyme is required for loop-mediated isothermal amplification (LAMP) of selected target nucleic acid sequences in a nucleic acid test (NAT).

The BST_LF_ was chosen as the polymerase, because of its DNA strand displacement functionality, thermostability and high processivity [22, 23]. The thermostability of BST_LF_ is advantageous when compared to other strand displacement polymerases (e.g. ϕ29, BSU) since LAMP is highly susceptible to primer dimers arising from the high primer count required in the mechanism [24]. Operating at higher temperatures with BST_LF_ reduces the likelihood of dimers, thereby reducing false positives. Furthermore, the high processivity of BST_LF_ ensures a higher output of targeted DNA amplification.

**Fig. 1:**
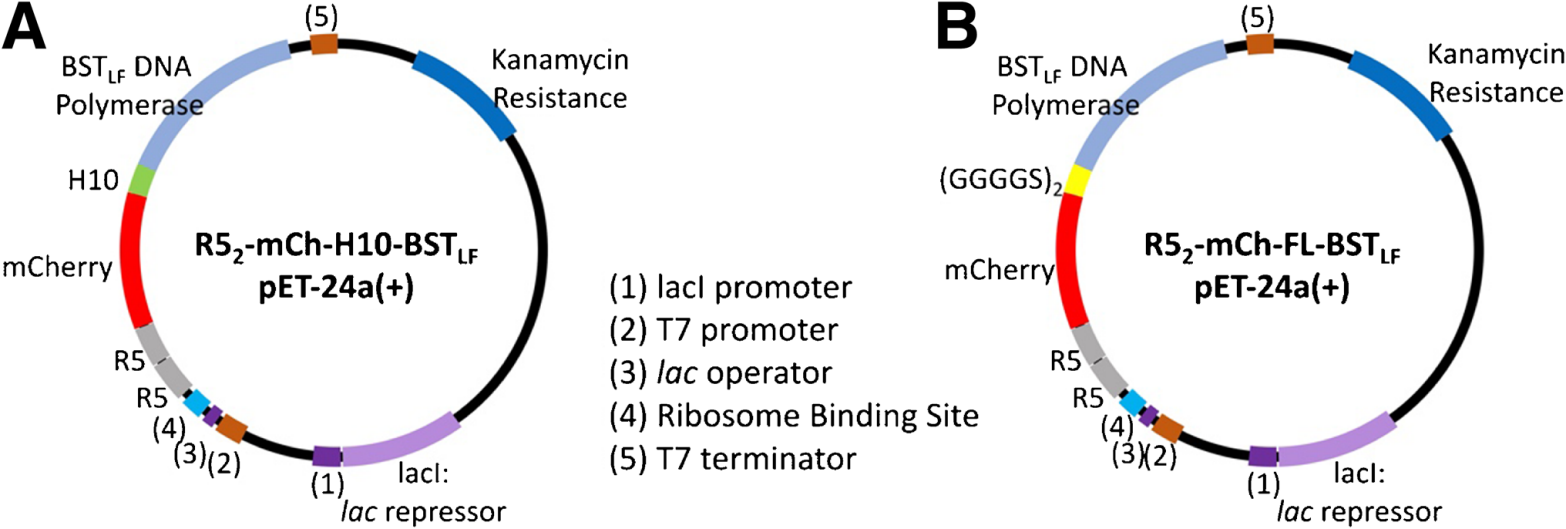
pET-24a( +) plasmid maps illustrating the N-terminal-modified BST_LF_ with **A** a long polyhistidine-tag (H10) and **B** a flexible linker (GGGGSGGGGS).

To enable comparison with more traditional His-tagged protein purification and isolation on a nickel-resin column, a long polyhistidine-tag (H10) sequence was also included in the fusion protein (R5_2_-mCh-H10-BST_LF_) construct (Fig. 1A) and comparison was made with a construct without H10 but containing a flexible linker (R5_2_-mCh-FL-BST_LF_, Fig. 1B). In both cases, only an N-terminal BST modification architecture was selected since C-terminal modifications disrupted the BST_LF_ activity (Figure S2).

### R5_2_-mCh-H10-BST_LF_ and R5_2_-mCh-FL-BST_LF_ expression

**Fig. 2:**
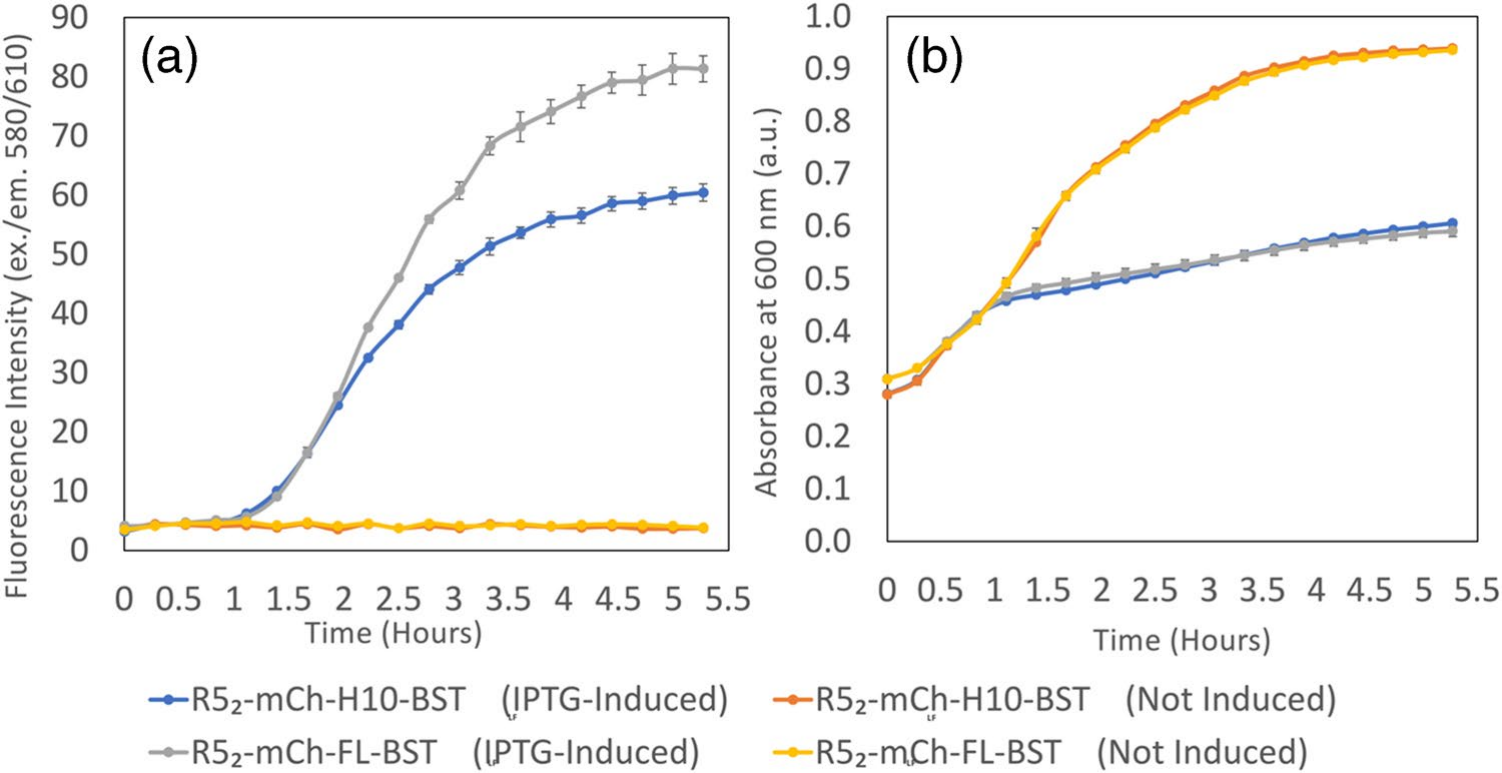
Protein expression via mCherry fluorescence (**A**) and absorbance at 600 nm due to *E. coli* cell growth (**B**) of the BL21 (DE3) for induced and non-induced cultures. One millimolar IPTG added to induce protein production at *t* = 0. Protein production begins 1 h after the induction

The mCherry was included in the construct as an in-built label. This is seen by comparing the difference in signal intensity due to mCherry fluorescence (Fig. 2A) and growth curve data (Fig. 2B) for protein-induced and non-induced cultures. As noted by others, high levels of protein expression following induction with 1 mM IPTG slow the bacterial growth (Fig. 2B) due to the metabolic burden on the cells during the protein expression and the probable toxic effect of IPTG [25, 26]. At 98–99 kDa, the significantly large fusion constructs, which were expressed in *E. coli*, yielded ~ 34.25 ± 1.90 mg/L and ~ 34.93 ± 2.63 mg/L of soluble protein (~ 38% and ~ 44% of total protein expressed) after 5 h of IPTG induction at 37 °C for R5_2_-mCh-H10-BST_LF_ and R5_2_-mCh-FL-BST_LF_, respectively.

The yield can be correlated with the in-built mCherry fluorescence and the production followed in real time (Fig. 2A). This enables easy production monitoring and product QA without more complex production systems. However, each construct needs independent calibration for mCherry fluorescence, according to the protein construct. There are a number of potential explanations for this construct-dependent mCherry fluorescence. Surface charge and local pI can play a role on the extinction coefficient for the chromophore and protein misfolding could impact the integrity of the chromophore, but the small decrease in fluorescence lifetime for the H10 construct compared with the FL construct (R5_2_-mCh-H10-BST_LF_: 1.587 ± 0.022 ns; R5_2_-mCh-FL-BST_LF_: 1.636 ± 0.08 ns at the same concentration) does not suggest that there is a significant change in the folding of the mCherry chromophore. Unlike the green fluorescent proteins, some of the red fluorescent proteins have reported a two-state model. In most cases, this refers to a second dark state, but in the case of mCherry, more than one brightness state has been proposed influenced by fusion partners at the C- or N-terminal, and a model with two brightness states described to explain the behaviour [27]. Thus, once the fusion construct mCherry fluorescence is calibrated for a particular system, it can be used to follow production and isolation.

### R5_2_-mCh-H10-BST_LF_ and R5_2_-mCh-FL-BST_LF_ purification

**Fig. 3:**
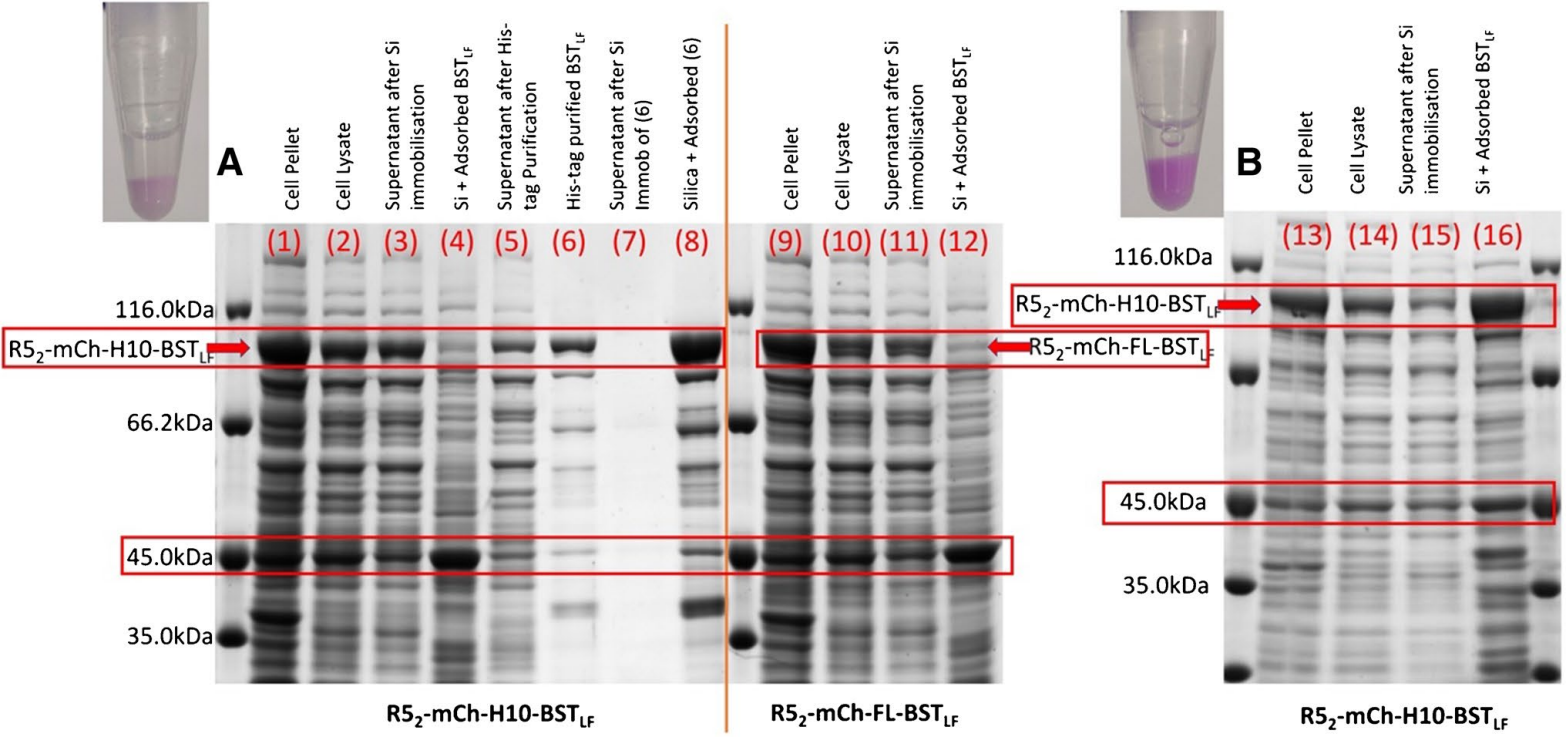
8% SDS-PAGE displaying R5_2_-mCh-H10-BST_LF_ (99.3kDA): **A** Immobilisation in Tris buffer (insert shows Eppendorf containing Si-R5_2_-mCh-H10-BST_LF_): (1) cell pellet, (2) cell lysate, (3) supernatant after silica adsorption, (4) silica protein desorption, post-protein adsorption, (5) supernatant after nickel-resin purification, (6) elution from nickel-resin, (7) supernatant after silica adsorption of nickel-resin elution, (8) silica post-nickel-resin elution adsorption. R5_2_-mCh-FL-BST_LF_ (98.6kDA): (9) cell pellet, (10) cell lysate, (11) supernatant after silica adsorption, (12) silica protein desorption, post-protein adsorption. **B** Eight percent SDS-PAGE highlighting PBS-based silica adsorption of R5_2_-mCh-H10-BST_LF_ (99.3kDA) (insert shows Eppendorf containing Si-R5_2_-mCh-H10-BST_LF_): (13) cell pellet, (14) cell lysate, (15) supernatant after silica adsorption, (16) silica protein desorption, post-protein adsorption

Protein adsorption onto the silica surface relies on electrostatic interaction [28]. The addition of the R5 19 amino acid peptide (SSKKSGSYSGSKGSKRRIL) to the construct shifts the pI of the fusion protein positive by ~ 1 unit for each R5 peptide unit added, rendering the fused protein more likely to bind to the negatively charged silica through the R5(s). From the pellet obtained from expression in *E. coli*, the strong band at around 98 kDa seen in the SDS-PAGE (Fig. 3, lane 1) can be attributed to R5_2_-mCh-H10-BST_LF_. Lane 2 shows the same band in the cell lysate and both lysate and pellet confirm that alongside the induced protein, many native *E. coli* proteins are present (as expected). Separation of the BST_LF_ proteins from these native proteins normally requires significant downstream processing steps, but the outcome downstream requirements have not been specified. In this work, the direct immobilisation of the R5 fusion construct with BST_LF_ onto silica was explored as a simple method for isolation of the BST_LF_ protein, and innovatively, we use the BST_LF_ in NATs still attached to the silica.

In evaluating this approach, of particular note from Fig. 3 is the prominent band at around 45 kDa: this is consistent with Elongation Factor-Tu, one of the most abundant native *E. coli* proteins [29, 30]. Lane 3 compares the lysate supernatant after it has been used for Si immobilisation with the protein bands that desorb from the Si after immobilisation and denaturation (lane 4). Measurement of the adsorption of the mCherry constructs on silica, through the characteristic absorption and fluorescence spectra, shows the residual band for R5_2_-mCh-H10-BST_LF_ on the SDS gel (lane 4) after denaturation, but the calculation of the difference between R5_2_-mCh-H10-BST_LF_ uptake from cell lysate and recovery after denaturation indicates that some of the R5_2_-mCh-H10-BST_LF_ remain tightly bound to the silica (~ 2 µg/mg protein/Si). This suggests very strong adhesion between the silaffin-tagged constructs and the silica particles, so that they remain on the silica, as well as the loosely bound proteins which are removed from the silica and migrate through the gel due to their electrophoretic mobility in the applied field.

If the R5_2_-mCh-H10-BST_LF_ is first purified from the cell lysate on a traditional histidine complexing Ni-resin (lane 6) before immobilisation on Si, the uptake of purified protein onto Si leaves only faint bands in the supernatant (lane 7), and an overload of the R5_2_-mCh-H10-BST_LF_ from the silica after denaturation (lane 8) shows that there is leaching of the R5_2_-mCh-H10-BST_LF_. Here, the difference between R5_2_-mCh-H10-BST_LF_ uptake from cell lysate and recovery after denaturation is ~ 20% higher at ~ 2.4 µg/g protein/Si. This partial desorption is reminiscent of the behaviour of another silaffin binding tag, Car9, where adsorption on silica is driven by cooperative binding [31]. When exposed to either cell lysate or purified protein, the primary layer of binding is both strong (irreversible) and selective to the R5 tags. The secondary layer of binding is weaker and non-selective. For the cell lysate, the most abundant protein after R5_2_-mCh-H10-BST_LF_ is the 45 kDa Elongation Factor-Tu, so the weaker secondary non-selective adsorption is dominated by this protein. This is reflected in the Si-desorption population profile in lane 4 and compares with similar behaviour for R5_2_-mCh-FL-BST_LF_ (Fig. 3 lanes 9–12). In contrast (lane 8) after Ni-resin purification, there are only trace concentrations of other proteins in the solution (mainly those containing histidine residues in the native *E. coli* proteins [32], lane 6), so the weaker secondary non-specific binding is dominated by more R5_2_-mCh-H10-BST_LF_ uptake from solution.

### R5_2_ fused BST protein immobilisation efficiency on silica

**Fig. 4:**
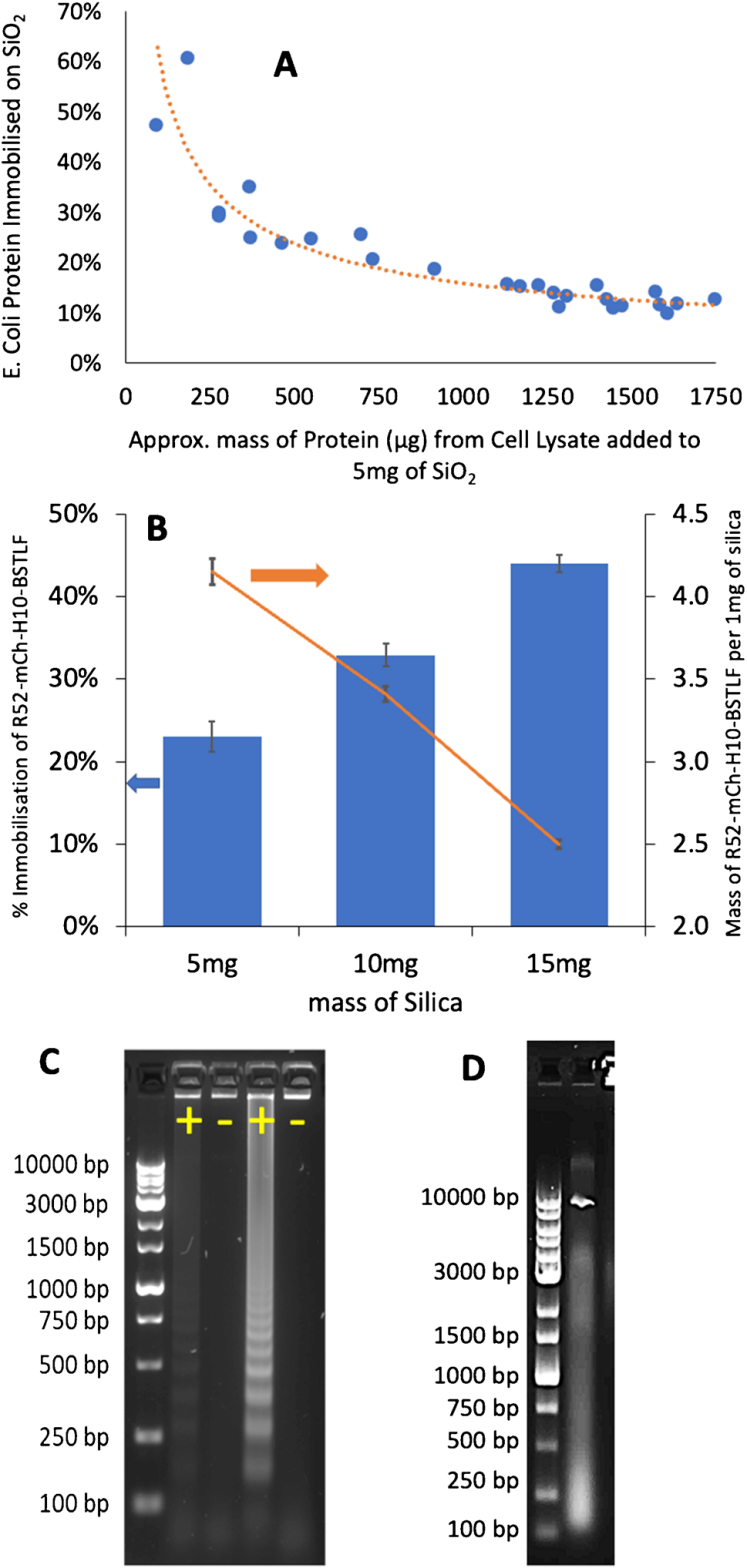
**A** Percentage of total protein binding from cell lysate to 5 mg of silica based on NanoDrop estimation of protein concentration in the lysate. **B** Comparison of R5_2_-mCh-H10-BST_LF_ loading efficiency (%) with the amount of BST_LF_/mg of silica (*n* = 3). **C** Agarose gel electrophoresis of LAMP products from 10^7^ copies of *P. malariae* 18S rRNA plasmid DNA using the P.MAL-LAU primer set (Table 1) and using 0.4 mg of silica with immobilised BST from cell lysate: lanes 1 and 2, 1250 µg of cell lysate + 15 mg of silica giving 2.5 µg/mg R5_2_-mCh-H10-BST_LF_/silica; lanes 3 and 4, 1250 µg of cell lysate + 5 mg of silica giving 4.15 µg/mg R5_2_-mCh-H10-BST_LF_/silica. LAMP was performed for 90 min. **D** Agarose gel electrophoresis of the DNA from the cell lysate (5 µL) containing R5_2_-mCh-H10-BST_LF_ showing the presence of native DNA fragments from 100–10,000 base pairs

Taking the cooperative binding on silica into account, the balance between the available silica surface and the R5 protein concentration in solution and uptake is important, to maximise the bound R5 construct. Figure 4A shows the overall loading of protein on silica according to the mass of cell lysate used. As expected, apparent efficiency is higher at lower concentrations of proteins in the cell lysate, but protein loading onto silica is more independent of cell lysate protein concentration at higher concentrations. From these data, 1250 µg cell lysate/5 mg silica gives a compromise between the maximum loading of the fusion protein and reproducibility between different production batches. Adjusting the ratio from 1250 µg cell lysate/5 mg silica to 1250 µg cell lysate/15 mg silica corresponds to lowering the loading from 4.15 to 2.5 µg of BST_LF_ per mg of silica. More protein/batch could be isolated using a higher silica to cell lysate ratio (Fig. 4B); however, this results in a lower local concentration of BST_LF_ around each silica particle and thus requires a higher volume of BST_LF_-silica to achieve the same number of units of enzyme for the nucleic acid test reaction. This can be illustrated in a LAMP reaction for *P. malariae* 18S rRNA plasmid DNA (Fig. 4C) using the P.MAL-LAU primer set (see Table 1). 0.4 mg of 2.5 µg/mg BST_LF_/silica produces only a faint positive result (lane 1) whereas 0.4 mg of 4.15 µg/mg of BST_LF_/silica produces a strong positive result (lane 3). Both BST_LF_-silica samples yielded true negative results (lanes 2 and 4), and the results also confirm that residual non-specific adsorption of native *E. coli* proteins on the silica, from the cell lysate, does not inhibit the LAMP reaction.

**Table 1:** Ninety-minute limit of detection comparison between LAMP run with commercial enzyme or recombinant enzyme depending on *Plasmodium* target and primer set (Lau et al. or Han et al.)

Malaria primer set	Limit of detection per reaction	
	Commercial BST_LF_	Si-R5_2_-mCh-FL-BST_LF_
18S rRNA (Lau et al.)		
*P. falciparum*	10^1^ copies^b^	No activity
*P. malariae*	10^4^ copies	No activity
*P. vivax*	10^1^ copies	10^5^ copies
*P. ovale*	10^5^ copies^b^	No activity
*P. knowlesi*	10^2^ copies	10^3^ copies
18S rRNA (Han et al.)		
*P. falciparum*	10^3^ copies^a^	10^2^ copies^a^
*P. vivax*	10^4^ copies^b^	10^3^ copies
*P. ovale*	10^5^ copies^b^	10^1^ copies
Mitochondrial DNA		
*P. falciparum*	10^2^ copies^a^	10^1^ copies^a^

^a^LoD test used genomic *P. falciparum* Dd2 DNA.

^b^Primer set had a high tendency for false positives.

Furthermore, importantly, the immobilisation of BST_LF_ on silica reduces the carryover of DNA from the cell lysate onto the silica and thereby reduces native background *E. coli* DNA contamination of the LAMP result. This can be shown using the same LAMP experiment but without the amplification target (i.e. negative conditions), comparing R5_2_-mCh-H10-BST_LF_ (Fig. 4C) in the raw cell lysate (5µL) without immobilisation on silica (Fig. 4D); it is clear that the raw cell lysate gives a high level of native background DNA (seen as a smear on the gel with the band at 10 K consistent with the pET24a( +) plasmid), which is not seen in Fig. 4C, lanes 2 and 4 with the R5_2_-mCh-H10-BST_LF_ on Si.

When scaling up the process, nearly identical levels of BST_LF_ absorption were observed on the silica for the 2 constructs with and without the H10 with yields of 4.38 ± 0.21 mg/L and 4.39 ± 1.23 mg/L of *E. coli* cell lysate for R5_2_-mCh-FL-BST_LF_ and R5_2_-mCh-H10-BST_LF_ respectively using 2 g silica. This indicates the conservation of the immobilisation domain. A higher amount of BST_LF_ (9.77 ± 0.09 mg/L) was isolated after nickel-resin purification. Considering the cooperative non-specific secondary binding mechanism, which will be dominated by BST_LF_ in this case, a higher loading is expected. Although a higher loading of BST_LF_ can be achieved on the silica with the extra Ni-resin purification step, it has to be considered whether this is essential. Both material and labour add a more significant cost for the Ni-resin column and reagents, compared with ≤ $0.25 for the silica/5000 tests (less if beach sand is used as the source of silica [12]). The direct Si isolation and immobilisation from cell lysate requires no additional chemicals or other reagents, providing a greener process. The Si immobilisation protocol automatically removes the transfer of the native background *E. coli* DNA contamination, but not all the native *E. coli* proteins, so it is necessary to establish how the resultant Si-R5_2_-mCh-FL-BST_LF_ and Si-R5_2_-mCh-H10-BST_LF_ compare for NAT use.

**Fig. 5:**
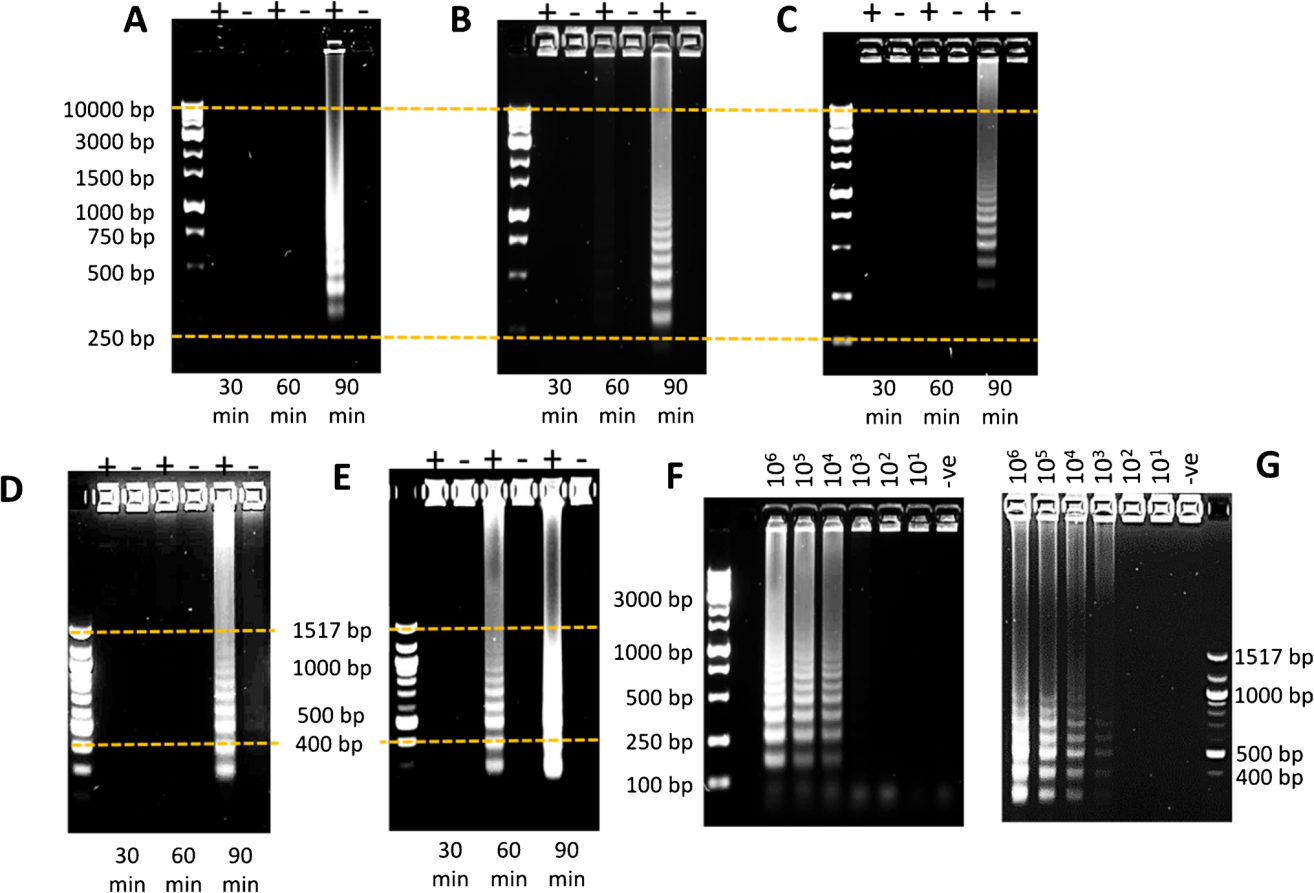
Comparative timed LAMP study targeting 10^6^ copies of *P. knowlesi* 18S rRNA using the P.KNO-LAU primer set with **A** 0.438 µg of nickel-purified R5_2_-mCh-H10-BST_LF_, **B** 0.878 µg of silica immobilised with R5_2_-mCh-H10-BST_LF_ from BST storage buffer and **C** 0.877 µg of silica immobilised with R5_2_-mCh-FL-BST_LF_ from BST storage buffer. LAMP reactivity comparison between R5_2_-mCh-H10-BST_LF_ immobilised in **D** BST storage buffer and **E** in PBS using 0.4 mg of Si-R5_2_-mCh-H10-BST_LF._ Limit of detection comparison between R5_2_-mCh-H10-BST immobilised on Si with **F** BST storage buffer and **G** PBS using 0.4 mg of Si-R5_2_-mCh-H10-BST_LF_

Both Si-R5_2_-mCh-FL-BST_LF_ (0.878 ± 0.245 µg) and Si-R5_2_-mCh-H10-BST_LF_ (0.877 ± 0.042 µg) were broadly comparable to nickel-purified R5_2_-mCh-H10-BST_LF_ (~ 0.438 µg) at half the equivalent solution concentration, when used in LAMP targeting 10^6^ copies of *P. knowlesi* 18S rRNA with the P.KNO-LAU primer set. This target/primer combination is more active than the target/primer set used in Fig. 4C, and as shown in Table 1 and Fig. 5C and D, at a lower concentration of Si-immobilised BST_LF_ (0.4 mg of 2.2 µg/mg of BST_LF_/silica = 0.878 µg BST_LF_), the amplified DNA could already be distinguished on the agarose gel after 60 min and revealed a more resolved LAMP banding pattern after 90 min.

### Improvement in R5_2_-fused BST protein immobilisation efficiency on silica

Clearly, there appears to be potential to improve the activity of the silica-immobilised BST_LF_. Phosphate buffer had been avoided in this immobilisation protocol, since phosphate can inhibit Taq polymerase and PCR [33, 34], possibly by binding to the phosphate-binding sites on the polymerase and inhibiting dNTP binding during the DNA synthesis reaction. However, if the Tris-based BST storage buffer used above was replaced with PBS during the immobilisation [15, 19, 35, 36], BST_LF_ adsorption onto silica increased to 14.33 ± 1.17 mg/L *E. coli* cell lysate (for 2 g silica). This outperforms the nickel-purified protein output (9.77 ± 0.09 mg/L) while still using only ≤ $0.25 worth of silica/5000 + NATs.

A study by Lechner et al. [37] proposed R5-silica precipitation is due to self-assembly via salt bridges between the guanidine group of a R5 arginine and the negatively charged phosphate ions. This could promote lateral cooperative secondary interactions as described above and improve the selectivity of the cooperative binding effect. As observed in Fig. 3B compared with Fig. 3A, the difference in the R5_2_-mCh-H10-BST_LF_ bands between lanes 14 and 15 (compared with the difference between lanes 2 and 3) highlights the improved uptake of the BST_LF_ protein onto the silica. Furthermore, the profile of the desorbed denatured protein population (lane 16) shows an increase in the BST_LF_ desorption band compared with the TRIS-based BST storage buffer immobilisation (Fig. 3A, lane 4). This is consistent with the secondary binding of the BST fusion protein and also shows a higher residual strongly attached R5_2_-mCh-H10-BST_LF_ on the Si (4 µg/mg BST_LF_/Si) which is not susceptible to denaturing desorption.

Having more immobilised protein per reaction (0.4 mg of 6.05 µg/mg of BST_LF_/silica = 2.419 µg BST_LF_) should increase the rate of reaction but it had a limited impact on the sensitivity. The gel images do not resolve sensitivity very accurately but do demonstrate that for the same primer set (P.KNO-LAU), the PBS-based Si-BST_LF_ required less time to amplify the same amount of target protein (Fig. 5E versus 5D). A stronger signal might also be expected with this PBS-immobilised enzyme for a lower copy number than the Tris buffer–immobilised enzyme, for a given primer set. However, in Fig. 5G (PBS immobilisation), a faint LAMP smear of DNA is seen at 10^3^ copies, but this is only slightly better than Fig. 5F (BST storage buffer immobilisation).

### Activity of the fusion proteins

**Fig. 6:**
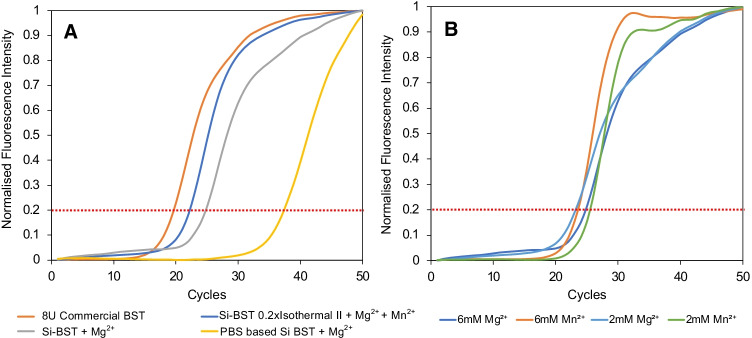
qPCR performed on output from BST_LF_ DNA amplification (15 min) targeting 100 copies of *P. knowlesi* using only the F3 and B3 primers from the P.KNO-LAU primer set. Activity assay, **A** (i) commercial BST_LF_ (8 U); (ii) Si-R5_2_-mCh-FL-BST_LF_ in ThermoPol® buffer with added Mg^2+^; (iii) 0.2 × isothermal amplification buffer with added Mn^2+^ and Mg^2+^; (iv) Si-R5_2_-mCh-FL-BST_LF_ immobilised in PBS and used in ThermoPol® buffer with added Mg^2+^. **B** Comparing the recombinant BST_LF_ on silica in ThermoPol® buffer, supplemented with different concentrations of Mn^2+^ and Mg^2+^. 1U of BST_LF_ is defined as the amount of enzyme required to incorporate 10 nmol of dNTP in 30 min at 65 °C.

Using quantitative nucleic acid amplification, the activity of the silica-immobilised enzymes can be more accurately compared than with the gels. This also allows the influence of certain dications to be explored, in the nucleic acid amplification, and optimised. Although quantitative LAMP (qLAMP) has been reported, the measurement of turbidity, due to the production of insoluble magnesium pyrophosphate as a by-product of the DNA synthesis, has a high error due to interference by the silica particles. The use of fluorescent labels (e.g. [38]) is also widely used and will be reported later with this Si/BST system, but as highlighted by others, these labels can also cause some inhibition of the enzyme. Thus, in order to compare the activity of the silica-immobilised BST_LF_ without additives, a two-step analysis was performed using a modified LAMP reaction targeting *P. knowlesi* 18S rRNA with only the F3/B3 primers from the P.KNO-LAU primer set (Figure S3). This reaction was run for 15 min and then the target DNA output was compared by running a real-time qPCR to obtain the threshold cycle (*C*_T_) number and calculate the activity compared with 8U commercial BST. Taking a threshold normalised fluorescence of 0.2, Fig. 6A shows the change in *C*_T_ values depending on the conditions used for the LAMP reaction, from which the apparent units of activity can be calculated (Table 2) from a set of calibration curves (Figure S3). The cell lysate containing (349 ± 26 ng) with a measured activity of 7.9 ± 1.0U had a similar DNA output within 15 min as found for 8U of commercial BST_LF_; however, the measured activity was dependent on the presence of certain divalent ions. For the DNA synthesis pathway, BST_LF_ polymerase needs to allow binding of β- and γ-phosphates of the dNTPs to strengthen the contact between the polymerase and its DNA substrates [39]. Association of the dNTPs and/or the conformational changes in the BST_LF_ that accompany the DNA synthesis mechanism and the release and breakdown of pyrophosphate (PPi) from the active site may become restricted in silica-immobilised BST_LF_. Furthermore, if the PPi is not properly released, it blocks the reaction and prevents the enzyme from moving to the next base. In solution-based polymerase-catalysed DNA synthesis, it has been proposed that the PPi release step can be improved by the addition of divalent ions that can complex the PPi [40, 41]. Mg^2+^ (2 mM) for example is used in the amplification medium, Thermopol®.

**Table 2:** Apparent units of BST_LF_ calculated from the qPCR performed on the output from LAMP (15 min) targeting 100 copies of *P. knowlesi* using the F3 and B3 primers from the P.KNO-LAU primer set. The LAMP reaction used (i) 8U commercial BST_LF_; (ii) cell lysate containing R5_2_-mCh-FL-BST_LF_ (349 ± 26 ng) or (iii) 0.4 mg of 2.19 µg/mg of R5_2_-mCh-FL-BST_LF_/silica. 1U of BST_LF_ is defined as the amount of enzyme required to incorporate 10 nmol of dNTP in 30 min at 65 °C

	Buffer*	Added [Mg^2+^]	Added [Mn^2+^]	*C*_T_ at 0.2	Units Activity seen
8U commercial BST_LF_	Thermopol®	6 mM		19.5 ± 0.5	8
Cell lysate with R5_2_-mCh-FL-BST_LF_	Thermopol®	6 mM		19.6 ± 0.3	7.9 ± 1.0
Si-R5_2_-mCh-FL-BST_LF_ immobilised from BST buffer	Thermopol®			33.6 ± 2.5	0.02 ± 0.002
	Thermopol® + Mg^2+^	2 mM		23.9 ± 1.3	1.7 ± 0.7
	Thermopol® + Mg^2+^	4 mM		24.9 ± 1.0	1.0 ± 0.4
	Thermopol® + Mg^2+^	6 mM		24.8 ± 0.4	0.8 ± 0.1
	Thermopol® + Mn^2+^		2 mM	25.5 ± 0.3	0.6 ± 0.06
	Thermopol® + Mn^2+^		4 mM	24.5 ± 0.1	0.9 ± 0.04
	Thermopol® + Mn^2+^		6 mM	23.6 ± 0.04	1.3 ± 0.02
	0.2 × Isothermal II (0.4 mM Mg^2+^) + Mn^2+^ + Mg^2+^	6 mM	1 mM	22.1 ± 0.2	2.4 ± 0.2
Si-R5_2_-mCh-FL-BST_LF_ immobilised from PBS buffer	Thermopol® + Mg^2+^	6 mM		38.9 ± 1.8	0.003 ± 0.002

*Thermopol® contains 2 mM Mg^2+^

Figure 6A shows the importance of phosphate in this mechanism: the *C*_T_ value resulting from the qPCR suggests a ~ 2^14^ lower DNA copy number emerging from the 15-min LAMP reaction, for the PBS-immobilised R5_2_-mCh-FL-BST_LF_, than for the immobilisation in phosphate-free BST buffer, using the same amount of R5_2_-mCh-FL-BST_LF_, under the same amplification conditions with 8 mM Mg^2+^. This would be consistent with phosphate ions blocking the sites for binding of β- and γ-phosphates of the dNTPs and may explain why the gels did not show improvement in sensitivity with the PBS-immobilised BST. On the other hand, comparing cell lysate activity or commercial BST_LF_ with the R5_2_-mCh-FL-BST_LF_ from phosphate-free BST buffer shows a ~ 2^5^ lower DNA copy number for the immobilised BST_LF_ under the same amplification conditions with 8 mM Mg^2+^. Noticeably, with just 2 mM Mg^2+^, the immobilised enzyme has very low activity (0.02 ± 0.002 Units) but this rises rapidly with Mg^2+^concentration to a maximum at 4 mM Mg^2+^ (1.7 ± 0.7 Units). Further increases in [Mg^2+^] do not increase activity. The polymerase synthesis is catalysed by two divalent metal ions in the nucleotidyl-transfer reaction. The metal ion plays a crucial role in lowering the p*K*_a_ of the 3′ hydroxyl group of the primer, thereby increasing its nucleophilicity for an attack on the α phosphorous atom of the incoming dNTP. However, the metal ion at the second binding site is associated with complexation with the pyrophosphate assisting in removing it from the binding site. The metal ion catalysis affects the affinity to a specific dNTP and incorporation of the correct dNMP, which is influenced by the choice of the metal ion. For example, there is a clear difference between [Mg^2+^] and [Mn^2+^]: whereas an increase in [Mg^2+^] by 2 mM above the background Thermopol® concentration increases the apparent activity of the BST_LF_ by > 80-fold, it requires the addition of 6 mM [Mn^2+^] to Thermopol® to increase the activity by a similar amount (Fig. 6B and Table 2). However, as reported by others, Mn^2+^ reduces the fidelity of the reaction [40, 41].

Nevertheless, by adding both Mg^2+^ (6 mM) and Mn^2+^ (1 mM) to the reaction mixture for DNA amplification, slightly improved *C*_T_ values could be obtained and thus the dication concentrations with the Si-R5_2_-mCh-FL-BST_LF_ immobilised from phosphate-free buffer have been optimised to high Mg^2+^ (6 mM) and low Mn^2+^ (1 mM), giving a *C*_T_ value that suggests that the LAMP output is only ~ 2^3^ lower than the 8U commercial BST_LF_. In terms of the sensitivity of the reaction, this is close to the commercial BST. Overall, a combination of [Mg^2+^] and [Mn^2+^] was found to provide the best outcome for LAMP targeting *P. knowlesi* 18S rRNA and was used for further testing.

### Evaluation of Si-R5_2_-mCh-FL-BST_LF_ for LAMP-based malaria diagnosis

Table 1 compares the limit of detection for different *Plasmodium* species and shows significant differences according to species and primer set. Despite the lower performance of Si-R5_2_-mCh-FL-BST_LF_ compared with the commercial BST_LF_ with the *P. knowlesi* 18S rRNA using the P.KNO-LAU primer set (Table 1), the Si-immobilised recombinant fusion protein outperformed the commercial enzyme for four of the other eight *Plasmodium* targeting LAMP primer sets tested.

The Lau et al. primer sets were generally not as active when using the Si-R5_2_-mCh-FL-BST_LF_ enzyme, but Si-R5_2_-mCh-FL-BST_LF_ showed higher sensitivity with the remaining primer sets and could consistently amplify as little as 10 copies per reaction in some cases. This is well within the WHO recommended target concentration of 200 parasites/µL for RDT evaluation [42]. Vashishtha et al. [41] reported on the role of the metal ion in base incorporation efficiency and an increase in the maximum rate constant of the pre-steady-state phase of nucleotide incorporation in the presence of Mn^2+^. In addition, better selectivity was observed with the silica-bound enzyme as primer-dimer amplification or non-template amplification (leading to false positives) was more often observed when using the commercial enzyme (Figure S4 and Figure S5). Although it remains unclear as to why one primer set was more sensitive than another despite targeting the same gene, a decrease in the fidelity of nucleotidyl transfer has been associated with the BST conformation and alignment of the triphosphate tail for catalysis, which is influenced by the divalent ion present and the particular base in the dNTP [41]. This may explain the difference found for different sequence primers.

It has been reported that using the online primer design software (Primer Explorer, used by both Lau et al. and Han et al.) is not enough to design a successful primer set [43, 44]. Without redesign and empirical testing, some of the generated primer sets can be hampered by low sensitivity and be prone to false positives [44]. This problem appears to stem from the fact that 4 to 6 primers need to be generated from a small segment of DNA (~ 250 bp). Considering the amount of restrictions that are applied to the primer design, automating the process is not trivial, and ideal targets/primer combinations might be missed [43]. Since the protein conformation and metal ion binding sites may be influenced by immobilisation, the differences between the performance of the primers will be expected to be directly associated with base-specific effects of the nucleotidyl-transfer mechanism, but these are insufficiently characterised to include in the primer design software.

**Fig. 7:**
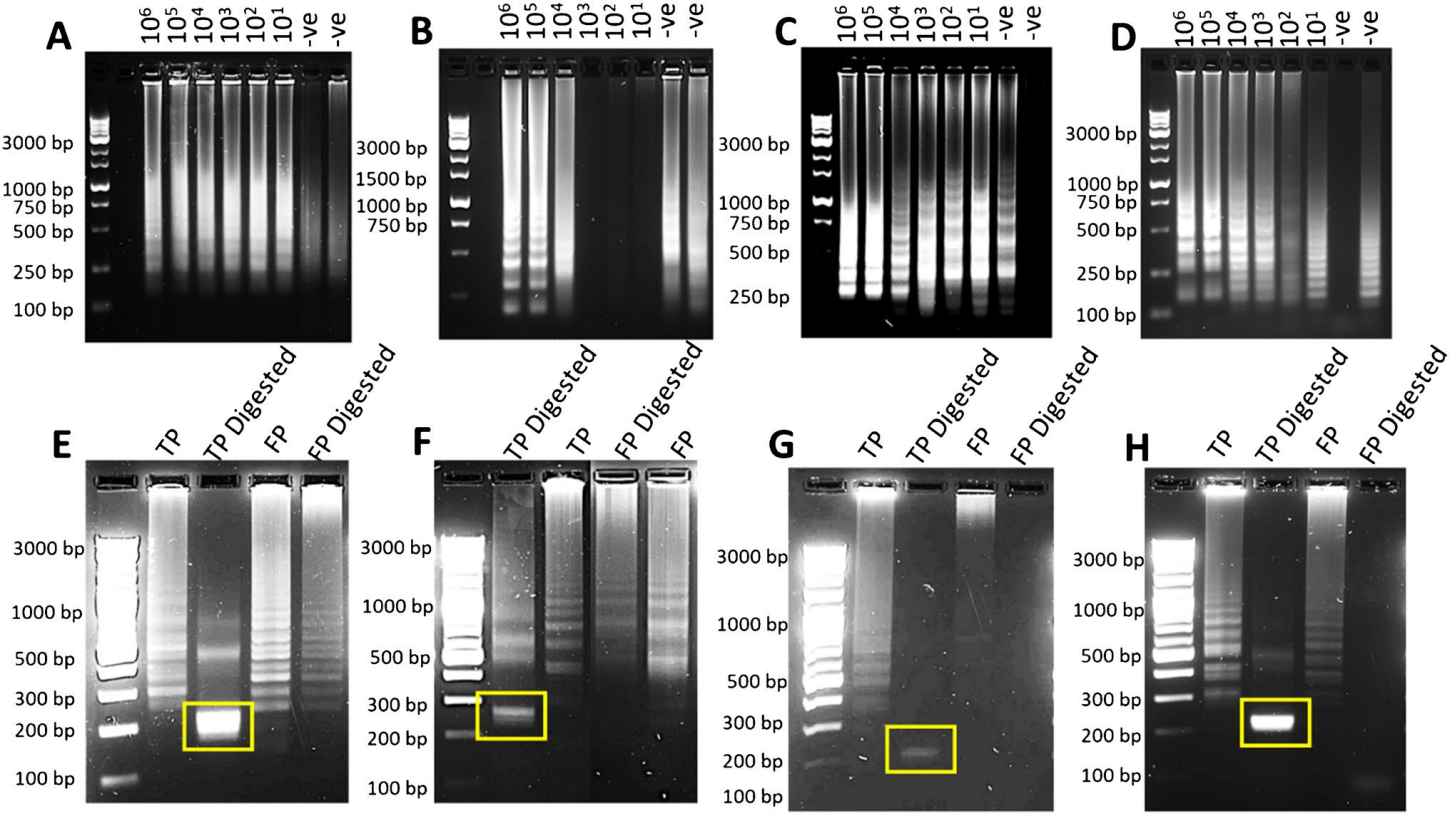
**A**–**D** 2% agarose gel electrophoresis of 90-min limit of detection LAMP using 8U of commercial BST_LF_ for the malaria primer sets showing false positives **A** P.FAL-LAU, **B** P.OVA-LAU, **C** P.VIV-HAN, **D** P.OVA-HAN. The number above each gel refers to the number of copies of plasmid DNA. **E**–**H** Restriction analysis of the true positive (TP) and false positive (FP). The products were run on a 2.5% agarose gel. **E** BbsI-HF® digestion of P.FAL-LAU products (expected band size of 224 bp). **F** BbsI-HF® digestion of P.OVA-LAU products (expected band size of 276 bp). **G** MfeI-HF® digestion of P.VIV-HAN products (expected band size of 219 bp). **H** MfeI-HF® digestion of P.OVA-HAN products (expected band size of 240 bp)

Certain malaria primer sets are more prone to primer-dimer amplification. For example, the following primer sets displayed a change in the band pattern of the LAMP output with low concentrations of the target DNA: P.FAL-LAU, P.OVA-LAU, P.VIV-HAN and P.OVA-HAN (Fig. 7A–D). This change in pattern may be the result of amplifiable primer dimers which “drive” the reaction by consuming the primers for non-specific amplification. To explore this further, restriction digest assays can distinguish the true LAMP amplification from the problematic primer amplification sets (Fig. 7E–H). The digested products of the true positives were in agreement with their predicted sizes, while the false positive products either did not digest (P.FAL-Lau and P.OVA-Lau) or were significantly smaller than the predicted sizes from a true positive (P.VIV-Han and P.OVA-Han). In the latter case, both the forward FIP primers contain the targeted digestion sequence (5’ – CAATTG – 3’). This could potentially indicate that the P.VIV-Han and P.OVA-Han FIP primers (Table S5) are at least partially responsible for the non-specific amplification observed in their respective assays. Similarly, the digestion sequence (5’ – GAAGAC – 3’) is present in the P.FAL-Lau BIP and P.OVA-Lau LPF primers. Since the false positive products from these primer sets did not digest, these primers are likely not to be responsible for the non-specific amplification in their respective assays.

### Production of Si-R5_2_-mCh-FL-BST_LF_ for field testing of samples for malaria testing

As discussed earlier, the Si-R5_2_-mCh-FL-BST_LF_ was designed with the aim to see whether the production of the enzyme could be easily transferred de novo to new sites and ultimately to low resource settings where the commercial enzyme is not affordable or to meet peak demands for local distributed diagnostic nucleic acid testing. Despite the higher enzyme loading using PBS for immobilisation on silica, the BST buffer–immobilised Si-R5_2_-mCh-FL-BST_LF_ was chosen for this initial trial, due to the potential inhibition of the nucleic acid amplification by phosphate from the PBS buffer indicated by the quantitative qPCR. Further work is needed to see whether this immobilisation protocol can be optimised to reduce residual phosphate from the immobilisation and take advantage of the higher BST loading.

As a first step to explore the transferability of production of R5_2_-mCh-FL-BST_LF_ for using nucleic acid tests, the plasmid designed at the University of Cambridge was sent to WACCBIP, the University of Ghana, to set up protein expression de novo. Using protocols derived in Cambridge, the plasmid was transformed and expressed using locally cultured *E. coli* and silica-based purification and immobilisation. To compare the performance of the Si-R5_2_-mCh-FL-BST_LF_ produced, the *P. falciparum* mitochondrial DNA primer set was used. Each production site showed a reproducible detection limit of 10 copies per reaction (without false positives), as shown in Figure S6. This was further referenced to production set up with colleagues at the Universiti Putra Malaysia (unpublished work), where the performance was also challenged at 5 copies per reaction, but at this level, it was not reliable (see Figure S6, UPM). The setup of expression and production of sufficient enzyme for the following trial was achieved de novo within 1 week.

### Clinical trial of P. falciparum detection in Ghana

Over 500 clinical samples were tested for *P. falciparum* using both the mitochondrial DNA and Han et al. primer sets, in comparison with PCR. The Lau et al. primer set was not chosen as it was prone to primer dimers producing false positives with the commercial BST enzyme and was not sufficiently active with the silica-immobilised enzyme.

The Si-R5_2_-mCh-FL-BST_LF_ LAMP assays produced a positive for *P. falciparum* for 277 out of 403 (69%) and 195 out of 393 (50%) PCR-positive samples when targeting the mitochondrial DNA and 18S rRNA gene, respectively (Table 3). “False positive” amplification (compared with PCR as reference) was observed with 15 of the 101 and 5 of the 97 PCR-negative samples, respectively. Assuming that the PCR is the gold standard, this leads to specificities of 85% for the mitochondrial DNA and 95% for the Han et al. primer sets, respectively.

**Table 3:** Comparison of the LAMP assays with PCR, targeting (i) mitochondrial DNA and (ii) 18S rRNA (using the Han et al. primer sets) for *P. falciparum*. Patient samples were collected from Cape Coast (CC), Asikuma (ASK) and Baiden-Ghartey Hospital (BGH)

Primer set comparison	Total sample population		CC sample population		ASK sample population		BGH sample population	
	PCR-positive	PCR-negative	PCR-positive	PCR-negative	PCR-positive	PCR-negative	PCR-positive	PCR-negative
Mitochondrial DNA (*n* = 504)								
Si-BST_LF_ positive	277	15	258	7	9	4	10	4
Si-BST_LF_ negative	126	86	86	70	0	0	40	16
18S rRNA (*n* = 490)								
Si-BST_LF_ positive	195	5	185	4	N/A	N/A	10	1
Si-BST_LF_ negative	198	92	158	73	N/A	N/A	40	19

The difference in sensitivity directly corresponds with the difference in the limit of detection between the two LAMP assays. As can be seen in Table 1, the mitochondrial DNA primer set has a lower detection limit than the Han et al. primer set for *P. falciparum*. Thus, a clinical sample that was only positive for the mitochondrial DNA LAMP assay would have a lower DNA copy number than those that tested positive for both primer sets. The positive qPCR results can thus be divided into 3 LAMP categories:

1. Testing positive for the mitochondrial DNA primer set and the 18S rRNA primer set
2. Testing positive for only the mitochondrial DNA primer set
3. Not testing positive with either primer set

This was further confirmed by comparing the qPCR threshold cycle (*C*_T_) number with the LAMP results (Table 4). The difference between the last two categories is just one cycle and since each qPCR amplification cycle yields a doubling in DNA, it suggests that the samples that did not yield a positive LAMP were only just below the detection threshold. One aspect of this is that the sensitivities of the LAMP assays varied between samples from different locations. For example, Cape Coast (CC) samples had higher LAMP sensitivity compared to those from Baiden-Ghartey Hospital (BGH), as shown in Table 3. Such geographical divergence suggests different protocols between centres, rather than a feature of the assay. Indeed, this was confirmed by local differences in the DNA extraction protocol used: for example, a larger elution volume used as standard for the BGH samples, potentially improving the overall DNA extraction yield, but this resulted in lower concentrations of target DNA than the CC samples, which used a smaller volume. Since the samples were originally intended for non-qPCR testing, the impact on the quantitative measurement only emerged in the assay development during the trial, but can assist in developing both the method and the protocol for the future (i.e. utilising smaller elution volumes to maximise the DNA concentration of each sample and potentially bringing more samples within the detection limits of the tested LAMP assay).

**Table 4:** Average qPCR cycles required for the detection of *P. falciparum* from positive samples detected by both LAMP assays (category 1), only the mitochondrial DNA LAMP assay (category 2) and neither LAMP assay (category 3)

Category	Result	Cycle threshold
1	+ P.FAL-MITO	21.2 ± 1.6
	+ P.FAL-HAN	
2	+ P.FAL-MITO	29.0 ± 0.7
	- P.FAL-HAN	
3	- P.FAL-MITO	30.4 ± 0.6
	- P.FAL-HAN	

## Conclusions

BST_LF_ fusion constructs were designed with an mCherry label and R5 silica-affinity tag, so that the polymerase enzyme could be expressed and isolated onto silica particles. The mCherry was found to be an efficient fluorescent and absorption label to follow the workflow from gene to diagnostic. Production of Si-R5_2_-mCh-FL-BST_LF_ and Si-R5_2_-mCh-H10-BST_LF_ was rapid and untroubled resulting in approximately 34mg of soluble protein after 5 h of IPTG induction. Despite the specific immobilisation of R5_2_-mCh-FL-BST_LF_ onto silica being accompanied by secondary weaker non-specific adsorption of native *E. coli* proteins, the Si-R5_2_-mCh-FL-BST_LF_ showed good activity and produced LAMP limits of detection within the WHO recommended target of 200 parasites/sample. The 75% sensitivity and 90% specificity WHO guideline was not tested at this concentration but specificities of 85% for the mitochondrial DNA and 95% for the 18s RNA Han et al. primer sets were obtained at a lower concentration. Mitochondrial DNA could be detected consistently down to 10 copies, whereas 18s RNA was detected at 100 copies.

From 1L cultures of *E. coli* cells, 4.38 ± 0.21 mg/L and 4.39 ± 1.23 mg/L of R5_2_-mCh-FL-BST_LF_ and R5_2_-mCh-H10-BST_LF_ respectively were immobilised on 2 g silica to provide ~ 5000 NATs. The amount of enzyme more than doubled, when immobilised in PBS buffer, but residual phosphate from the buffer, possibly blocking the sites on the BST_LF_ for binding of β- and γ-phosphates of the dNTPs, reduced the activity of the protein. The quantitative assessment of the enzyme activity showed the importance of Mg^2+^ and Mn^2+^ in optimising the enzyme activity. These cations complex the PPi product from the polymerisation aiding its release from the BST_LF_ and the progression of the enzyme to the next base.

Si-R5_2_-mCh-FL-BST_LF_ was further tested with different species of *Plasmodium,* and interestingly the detection limit in comparison with the commercial BST_LF_ depended on both species and primer set. The lower performance of Si-R5_2_-mCh-FL-BST_LF_ compared with the commercial BST_LF_ with the *P. knowlesi* 18S rRNA using the P.KNO-LAU primer set was seen, but the Si-immobilised recombinant fusion protein outperformed the commercial enzyme for four of the other eight *Plasmodium* targeting LAMP primer sets tested. It was also noticed that the Lau et al. primer sets were generally not as active for the Si-immobilised protein. It was not clear why these different primer sets showed such different performances. They also showed different tendencies towards the primer-dimer formation, which may be related to this, as well as influencing the likelihood of false positives. These could be identified by a digest of the amplification product and comparison of the size of the DNA band in comparison with the true positive.

The Si-R5_2_-mCh-FL-BST_LF_ was easy to produce; the expression of R5_2_-mCh-FL-BST_LF_ was transferred from Cambridge to Accra and set up de novo to generate sufficient Si-R5_2_-mCh-FL-BST_LF_ for a trial with clinical samples within 1 week. The analysis of samples for *P. falciparum* showed that the Si-R5_2_-mCh-FL-BST_LF_ had a different detection limit targeting the mitochondrial DNA or the 18S rRNA gene in a manner consistent with the qPCR results and also revealed the importance of sampling protocol in the test outcomes. Assuming a “true” result from the gold standard PCR assay, this provides promise that the Si-BST polymerase could be optimised to meet the WHO recommended guidelines and produced locally as part of the diagnostic manufacturing process at circa 5% of the cost of the current BST enzyme in LMICs.

## Supplementary Information

Below is the link to the electronic supplementary material.

Supplementary file1 (DOCX 2.93 MB)
