# Supplementary material for: Analysis and validation of silica-immobilised BST polymerase in loop-mediated isothermal amplification (LAMP) for malaria diagnosis
Source: Anal Bioanal Chem. 2022 Jun 3;414(21):6309–26. doi: 10.1007/s00216-022-04131-2 (PMC9163865; doi:10.1007/s00216-022-04131-2)
Supplement: Supplementary file 1 — Supplementary file1 (DOCX 2.93 MB) [file 216_2022_4131_MOESM1_ESM.docx]

**Analysis and validation of silica immobilised BST polymerase in loop-mediated isothermal amplification (LAMP) for malaria diagnosis.**

Dushanth Seevaratnam^a^, Felix Ansah^b^, Yaw Aniweh^b^, Gordon A Awandare^b^, Elizabeth A. H. Hall^a^*

*^a^* Department of Chemical Engineering and Biotechnology, University of Cambridge, Philippa Fawcett Drive, Cambridge, CB3 0AS, UK

*^b^* West African Centre for Cell Biology of Infectious Pathogens, University of Ghana, P.O. Box Lg 54, Legon- Accra

*corresponding author: eah16@cam.ac.uk

**Supporting Figures and Tables**

Table S1: List of Primers used to generate DNA fragments for Klenow Assembly

Table S2: Nucleotide and amino acid sequences for protein constructs studied

| **DNA Name** | **Nucleotide Sequence** |
| --- | --- |
| Flexible Linker | GGAGGTGGCGGATCGGGAGGTGGCGGATCG |
| R5 peptide | TCCTCTAAAAAGTCTGGTTCCTACTCTGGTAGCAAAGGCTCCAAACGTCGCATCCTG |
| mCherry | GTGAGCAAGGGCGAGGAGGATAACATGGCCATCATCAAGGAGTTCATGCGCTTCAAGGTGCACATGGAGGGCTCCGTGAACGGCCACGAGTTCGAGATCGAGGGCGAGGGCGAGGGCCGCCCCTACGAGGGCACCCAGACCGCCAAGCTGAAGGTGACCAAGGGTGGCCCCCTGCCCTTCGCCTGGGACATCCTGTCCCCTCAGTTCATGTACGGCTCCAAGGCCTACGTGAAGCACCCCGCCGACATCCCCGACTACTTGAAGCTGTCCTTCCCCGAGGGCTTCAAGTGGGAGCGCGTGATGAACTTCGAGGACGGCGGCGTGGTGACCGTGACCCAGGACTCCTCCCTGCAGGACGGCGAGTTCATCTACAATGTGAAGCTGCGCGGCACCAACTTCCCCTCCGACGGCCCCGTAATGCAGAAGAAGACCATGGGCTGGGAGGCCTCCTCCGAGCGGATGTACCCCGAGGACGGCGCCCTGAAGGGCGAGATCAAGCAGAGGCTGAAGCTGAAGGACGGCGGCCACTACGACGCTGAGGTCAAGACCACCTACAAGGCCAAGAAGCCCGTGCAGCTGCCCGGCGCCTACAACGTCAACATCAAGTTGGACATCACCTCCCACAACGAGGACTACACCATCGTGGAACAGTACGAACGCGCCGAGGGCCGCCACTCCACCGGCGGCATGGACGAGCTGTACAAGGGATCC |
| BST_LF_ | ACGGATGAAGGCGAAAAGCCGCTCGCCGGGATGGATTTTGCGATCGCCGACAGCGTCACGGACGAAATGCTCGCCGACAAAGCGGCCCTCGTCGTGGAGGTGGTGGGCGACAACTATCACCATGCCCCGATTGTCGGGATCGCCTTGGCCAACGAACGCGGGCGGTTTTTCCTGCGCCCGGAGACGGCGCTCGCCGATCCGAAATTTCTCGCTTGGCTTGGCGATGAGACGAAGAAAAAAACGATGTTTGATTCAAAGCGGGCGGCCGTCGCGCTAAAATGGAAAGGAATCGAACTGCGCGGCGTCGTGTTCGATCTGTTGCTGGCCGCTTACTTGCTCGATCCGGCGCAGGCGGCGGGCGACGTTGCCGCGGTGGCGAAAATGCATCAGTACGAGGCGGTGCGATCGGATGAGGCGGTCTATGGAAAAGGAGCGAAGCGGACGGTTCCTGATGAACCGACGCTTGCCGAGCATCTCGCCCGCAAGGCGGCGGCCATTTGGGCGCTTGAAGAGCCGTTGATGGACGAACTGCGCCGCAACGAACAAGATCGGCTGCTGACCGAGCTCGAACAGCCGCTGGCTGGCATTTTGGCCAATATGGAATTTACTGGAGTGAAAGTGGACACGAAGCGGCTTGAACAGATGGGGGCGGAGCTCACCGAGCAGCTGCAGGCGGTCGAGCGGCGCATTTACGAACTCGCCGGCCAAGAGTTCAACATTAACTCGCCGAAACAGCTCGGGACGGTTTTATTTGACAAGCTGCAGCTCCCGGTGTTGAAAAAGACAAAAACCGGCTATTCGACTTCAGCCGATGTGCTTGAGAAGCTTGCACCGCACCATGAAATCGTCGAACATATTTTGCATTACCGCCAACTCGGCAAGCTGCAGTCAACGTATATTGAAGGGCTGCTGAAAGTGGTGCACCCCGTGACGGGCAAAGTGCACACGATGTTCAATCAGGCGTTGACGCAAACCGGGCGCCTCAGCTCCGTCGAACCGAATTTGCAAAACATTCCGATTCGGCTTGAGGAAGGGCGGAAAATCCGCCAGGCGTTCGTGCCGTCGGAGCCGGACTGGCTCATCTTTGCGGCCGACTATTCGCAAATCGAGCTGCGCGTCCTCGCCCATATCGCGGAAGATGACAATTTGATTGAAGCGTTCCGGCGCGGGTTGGACATCCATACGAAAACAGCCATGGACATTTTCCATGTGAGCGAAGAAGACGTGACAGCCAACATGCGCCGCCAAGCGAAGGCCGTCAATTTTGGCATCGTGTACGGCATTAGTGATTACGGTCTGGCGCAAAACTTGAACATTACGCGCAAAGAAGCGGCTGAATTTATTGAGCGATATTTTGCCAGTTTTCCAGGTGTAAAGCAATATATGGACAACATTGTGCAAGAAGCGAAACAAAAAGGGTATGTGACGACGCTGCTGCATCGGCGCCGCTATTTGCCCGATATTACAAGCCGCAACTTCAACGTCCGCAGCTTCGCCGAGCGGACGGCGATGAACACACCGATCCAAGGGAGTGCCGCTGATATTATTAAAAAAGCGATGATCGATCTAAGCGTGAGGCTGCGCGAAGAACGGCTGCAGGCGCGCCTGTTGCTGCAAGTGCATGACGAACTCATTTTGGAGGCGCCGAAAGAGGAAATCGAGCGGCTGTGCCGCCTCGTTCCAGAGGTGATGGAGCAAGCCGTCGCACTCCGCGTGCCGCTGAAAGTCGATTACCATTACGGTCCGACGTGGTACGACGCCAAA |
| R5_2_-mCh-H10-BST_LF_ | ATGGGATCCTCCTCTAAAAAGTCTGGTTCCTACTCTGGTAGCAAAGGCTCCAAACGTCGCATCCTGTCCTCTAAAAAGTCTGGTTCCTACTCTGGTAGCAAAGGCTCCAAACGTCGCATCCTGAATTTAGTGAGCAAGGGCGAGGAGGATAACATGGCCATCATCAAGGAGTTCATGCGCTTCAAGGTGCACATGGAGGGCTCCGTGAACGGCCACGAGTTCGAGATCGAGGGCGAGGGCGAGGGCCGCCCCTACGAGGGCACCCAGACCGCCAAGCTGAAGGTGACCAAGGGTGGCCCCCTGCCCTTCGCCTGGGACATCCTGTCCCCTCAGTTCATGTACGGCTCCAAGGCCTACGTGAAGCACCCCGCCGACATCCCCGACTACTTGAAGCTGTCCTTCCCCGAGGGCTTCAAGTGGGAGCGCGTGATGAACTTCGAGGACGGCGGCGTGGTGACCGTGACCCAGGACTCCTCCCTGCAGGACGGCGAGTTCATCTACAATGTGAAGCTGCGCGGCACCAACTTCCCCTCCGACGGCCCCGTAATGCAGAAGAAGACCATGGGCTGGGAGGCCTCCTCCGAGCGGATGTACCCCGAGGACGGCGCCCTGAAGGGCGAGATCAAGCAGAGGCTGAAGCTGAAGGACGGCGGCCACTACGACGCTGAGGTCAAGACCACCTACAAGGCCAAGAAGCCCGTGCAGCTGCCCGGCGCCTACAACGTCAACATCAAGTTGGACATCACCTCCCACAACGAGGACTACACCATCGTGGAACAGTACGAACGCGCCGAGGGCCGCCACTCCACCGGCGGCATGGACGAGCTGTACAAGCACCACCATCATCACCATCACCATCACCACGGAGGTGGCTCGAATTCGACGGATGAAGGCGAAAAGCCGCTCGCCGGGATGGATTTTGCGATCGCCGACAGCGTCACGGACGAAATGCTCGCCGACAAAGCGGCCCTCGTCGTGGAGGTGGTGGGCGACAACTATCACCATGCCCCGATTGTCGGGATCGCCTTGGCCAACGAACGCGGGCGGTTTTTCCTGCGCCCGGAGACGGCGCTCGCCGATCCGAAATTTCTCGCTTGGCTTGGCGATGAGACGAAGAAAAAAACGATGTTTGATTCAAAGCGGGCGGCCGTCGCGCTAAAATGGAAAGGAATCGAACTGCGCGGCGTCGTGTTCGATCTGTTGCTGGCCGCTTACTTGCTCGATCCGGCGCAGGCGGCGGGCGACGTTGCCGCGGTGGCGAAAATGCATCAGTACGAGGCGGTGCGATCGGATGAGGCGGTCTATGGAAAAGGAGCGAAGCGGACGGTTCCTGATGAACCGACGCTTGCCGAGCATCTCGCCCGCAAGGCGGCGGCCATTTGGGCGCTTGAAGAGCCGTTGATGGACGAACTGCGCCGCAACGAACAAGATCGGCTGCTGACCGAGCTCGAACAGCCGCTGGCTGGCATTTTGGCCAATATGGAATTTACTGGAGTGAAAGTGGACACGAAGCGGCTTGAACAGATGGGGGCGGAGCTCACCGAGCAGCTGCAGGCGGTCGAGCGGCGCATTTACGAACTCGCCGGCCAAGAGTTCAACATTAACTCGCCGAAACAGCTCGGGACGGTTTTATTTGACAAGCTGCAGCTCCCGGTGTTGAAAAAGACAAAAACCGGCTATTCGACTTCAGCCGATGTGCTTGAGAAGCTTGCACCGCACCATGAAATCGTCGAACATATTTTGCATTACCGCCAACTCGGCAAGCTGCAGTCAACGTATATTGAAGGGCTGCTGAAAGTGGTGCACCCCGTGACGGGCAAAGTGCACACGATGTTCAATCAGGCGTTGACGCAAACCGGGCGCCTCAGCTCCGTCGAACCGAATTTGCAAAACATTCCGATTCGGCTTGAGGAAGGGCGGAAAATCCGCCAGGCGTTCGTGCCGTCGGAGCCGGACTGGCTCATCTTTGCGGCCGACTATTCGCAAATCGAGCTGCGCGTCCTCGCCCATATCGCGGAAGATGACAATTTGATTGAAGCGTTCCGGCGCGGGTTGGACATCCATACGAAAACAGCCATGGACATTTTCCATGTGAGCGAAGAAGACGTGACAGCCAACATGCGCCGCCAAGCGAAGGCCGTCAATTTTGGCATCGTGTACGGCATTAGTGATTACGGTCTGGCGCAAAACTTGAACATTACGCGCAAAGAAGCGGCTGAATTTATTGAGCGATATTTTGCCAGTTTTCCAGGTGTAAAGCAATATATGGACAACATTGTGCAAGAAGCGAAACAAAAAGGGTATGTGACGACGCTGCTGCATCGGCGCCGCTATTTGCCCGATATTACAAGCCGCAACTTCAACGTCCGCAGCTTCGCCGAGCGGACGGCGATGAACACACCGATCCAAGGGAGTGCCGCTGATATTATTAAAAAAGCGATGATCGATCTAAGCGTGAGGCTGCGCGAAGAACGGCTGCAGGCGCGCCTGTTGCTGCAAGTGCATGACGAACTCATTTTGGAGGCGCCGAAAGAGGAAATCGAGCGGCTGTGCCGCCTCGTTCCAGAGGTGATGGAGCAAGCCGTCGCACTCCGCGTGCCGCTGAAAGTCGATTACCATTACGGTCCGACGTGGTACGACGCCAAATGA |
| R5_2_-mCh-FL-BST_LF_ | ATGGGATCCTCCTCTAAAAAGTCTGGTTCCTACTCTGGTAGCAAAGGCTCCAAACGTCGCATCCTGTCCTCTAAAAAGTCTGGTTCCTACTCTGGTAGCAAAGGCTCCAAACGTCGCATCCTGAATTTAGTGAGCAAGGGCGAGGAGGATAACATGGCCATCATCAAGGAGTTCATGCGCTTCAAGGTGCACATGGAGGGCTCCGTGAACGGCCACGAGTTCGAGATCGAGGGCGAGGGCGAGGGCCGCCCCTACGAGGGCACCCAGACCGCCAAGCTGAAGGTGACCAAGGGTGGCCCCCTGCCCTTCGCCTGGGACATCCTGTCCCCTCAGTTCATGTACGGCTCCAAGGCCTACGTGAAGCACCCCGCCGACATCCCCGACTACTTGAAGCTGTCCTTCCCCGAGGGCTTCAAGTGGGAGCGCGTGATGAACTTCGAGGACGGCGGCGTGGTGACCGTGACCCAGGACTCCTCCCTGCAGGACGGCGAGTTCATCTACAATGTGAAGCTGCGCGGCACCAACTTCCCCTCCGACGGCCCCGTAATGCAGAAGAAGACCATGGGCTGGGAGGCCTCCTCCGAGCGGATGTACCCCGAGGACGGCGCCCTGAAGGGCGAGATCAAGCAGAGGCTGAAGCTGAAGGACGGCGGCCACTACGACGCTGAGGTCAAGACCACCTACAAGGCCAAGAAGCCCGTGCAGCTGCCCGGCGCCTACAACGTCAACATCAAGTTGGACATCACCTCCCACAACGAGGACTACACCATCGTGGAACAGTACGAACGCGCCGAGGGCCGCCACTCCACCGGCGGCATGGACGAGCTGTACAAGGGAGGTGGCGGATCGGGAGGTGGCGGATCGGGAGGTGGCTCGAATTCGACGGATGAAGGCGAAAAGCCGCTCGCCGGGATGGATTTTGCGATCGCCGACAGCGTCACGGACGAAATGCTCGCCGACAAAGCGGCCCTCGTCGTGGAGGTGGTGGGCGACAACTATCACCATGCCCCGATTGTCGGGATCGCCTTGGCCAACGAACGCGGGCGGTTTTTCCTGCGCCCGGAGACGGCGCTCGCCGATCCGAAATTTCTCGCTTGGCTTGGCGATGAGACGAAGAAAAAAACGATGTTTGATTCAAAGCGGGCGGCCGTCGCGCTAAAATGGAAAGGAATCGAACTGCGCGGCGTCGTGTTCGATCTGTTGCTGGCCGCTTACTTGCTCGATCCGGCGCAGGCGGCGGGCGACGTTGCCGCGGTGGCGAAAATGCATCAGTACGAGGCGGTGCGATCGGATGAGGCGGTCTATGGAAAAGGAGCGAAGCGGACGGTTCCTGATGAACCGACGCTTGCCGAGCATCTCGCCCGCAAGGCGGCGGCCATTTGGGCGCTTGAAGAGCCGTTGATGGACGAACTGCGCCGCAACGAACAAGATCGGCTGCTGACCGAGCTCGAACAGCCGCTGGCTGGCATTTTGGCCAATATGGAATTTACTGGAGTGAAAGTGGACACGAAGCGGCTTGAACAGATGGGGGCGGAGCTCACCGAGCAGCTGCAGGCGGTCGAGCGGCGCATTTACGAACTCGCCGGCCAAGAGTTCAACATTAACTCGCCGAAACAGCTCGGGACGGTTTTATTTGACAAGCTGCAGCTCCCGGTGTTGAAAAAGACAAAAACCGGCTATTCGACTTCAGCCGATGTGCTTGAGAAGCTTGCACCGCACCATGAAATCGTCGAACATATTTTGCATTACCGCCAACTCGGCAAGCTGCAGTCAACGTATATTGAAGGGCTGCTGAAAGTGGTGCACCCCGTGACGGGCAAAGTGCACACGATGTTCAATCAGGCGTTGACGCAAACCGGGCGCCTCAGCTCCGTCGAACCGAATTTGCAAAACATTCCGATTCGGCTTGAGGAAGGGCGGAAAATCCGCCAGGCGTTCGTGCCGTCGGAGCCGGACTGGCTCATCTTTGCGGCCGACTATTCGCAAATCGAGCTGCGCGTCCTCGCCCATATCGCGGAAGATGACAATTTGATTGAAGCGTTCCGGCGCGGGTTGGACATCCATACGAAAACAGCCATGGACATTTTCCATGTGAGCGAAGAAGACGTGACAGCCAACATGCGCCGCCAAGCGAAGGCCGTCAATTTTGGCATCGTGTACGGCATTAGTGATTACGGTCTGGCGCAAAACTTGAACATTACGCGCAAAGAAGCGGCTGAATTTATTGAGCGATATTTTGCCAGTTTTCCAGGTGTAAAGCAATATATGGACAACATTGTGCAAGAAGCGAAACAAAAAGGGTATGTGACGACGCTGCTGCATCGGCGCCGCTATTTGCCCGATATTACAAGCCGCAACTTCAACGTCCGCAGCTTCGCCGAGCGGACGGCGATGAACACACCGATCCAAGGGAGTGCCGCTGATATTATTAAAAAAGCGATGATCGATCTAAGCGTGAGGCTGCGCGAAGAACGGCTGCAGGCGCGCCTGTTGCTGCAAGTGCATGACGAACTCATTTTGGAGGCGCCGAAAGAGGAAATCGAGCGGCTGTGCCGCCTCGTTCCAGAGGTGATGGAGCAAGCCGTCGCACTCCGCGTGCCGCTGAAAGTCGATTACCATTACGGTCCGACGTGGTACGACGCCAAATGA |

| **Peptide Name** | **Amino Acid Sequence** |
| --- | --- |
| Flexible Linker | GGGGSGGGGS |
| R5 peptide | SSKKSGSYSGSKGSKRRIL |
| mCherry | VSKGEEDNMAIIKEFMRFKVHMEGSVNGHEFEIEGEGEGRPYEGTQTAKLKVTKGGPLPFAWDILSPQFMYGSKAYVKHPADIPDYLKLSFPEGFKWERVMNFEDGGVVTVTQDSSLQDGEFIYNVKLRGTNFPSDGPVMQKKTMGWEASSERMYPEDGALKGEIKQRLKLKDGGHYDAEVKTTYKAKKPVQLPGAYNVNIKLDITSHNEDYTIVEQYERAEGRHSTGGMDELYK |
| BST_LF_ | TDEGEKPLAGMDFAIADSVTDEMLADKAALVVEVVGDNYHHAPIVGIALANERGRFFLRPETALADPKFLAWLGDETKKKTMFDSKRAAVALKWKGIELRGVVFDLLLAAYLLDPAQAAGDVAAVAKMHQYEAVRSDEAVYGKGAKRTVPDEPTLAEHLARKAAAIWALEEPLMDELRRNEQDRLLTELEQPLAGILANMEFTGVKVDTKRLEQMGAELTEQLQAVERRIYELAGQEFNINSPKQLGTVLFDKLQLPVLKKTKTGYSTSADVLEKLAPHHEIVEHILHYRQLGKLQSTYIEGLLKVVHPVTGKVHTMFNQALTQTGRLSSVEPNLQNIPIRLEEGRKIRQAFVPSEPDWLIFAADYSQIELRVLAHIAEDDNLIEAFRRGLDIHTKTAMDIFHVSEEDVTANMRRQAKAVNFGIVYGISDYGLAQNLNITRKEAAEFIERYFASFPGVKQYMDNIVQEAKQKGYVTTLLHRRRYLPDITSRNFNVRSFAERTAMNTPIQGSAADIIKKAMIDLSVRLREERLQARLLLQVHDELILEAPKEEIERLCRLVPEVMEQAVALRVPLKVDYHYGPTWYDAK |
| R5_2_-mCh-H10-BST_LF_ | MGSSSKKSGSYSGSKGSKRRILSSKKSGSYSGSKGSKRRILNLVSKGEEDNMAIIKEFMRFKVHMEGSVNGHEFEIEGEGEGRPYEGTQTAKLKVTKGGPLPFAWDILSPQFMYGSKAYVKHPADIPDYLKLSFPEGFKWERVMNFEDGGVVTVTQDSSLQDGEFIYNVKLRGTNFPSDGPVMQKKTMGWEASSERMYPEDGALKGEIKQRLKLKDGGHYDAEVKTTYKAKKPVQLPGAYNVNIKLDITSHNEDYTIVEQYERAEGRHSTGGMDELYKHHHHHHHHHHGGGSNSTDEGEKPLAGMDFAIADSVTDEMLADKAALVVEVVGDNYHHAPIVGIALANERGRFFLRPETALADPKFLAWLGDETKKKTMFDSKRAAVALKWKGIELRGVVFDLLLAAYLLDPAQAAGDVAAVAKMHQYEAVRSDEAVYGKGAKRTVPDEPTLAEHLARKAAAIWALEEPLMDELRRNEQDRLLTELEQPLAGILANMEFTGVKVDTKRLEQMGAELTEQLQAVERRIYELAGQEFNINSPKQLGTVLFDKLQLPVLKKTKTGYSTSADVLEKLAPHHEIVEHILHYRQLGKLQSTYIEGLLKVVHPVTGKVHTMFNQALTQTGRLSSVEPNLQNIPIRLEEGRKIRQAFVPSEPDWLIFAADYSQIELRVLAHIAEDDNLIEAFRRGLDIHTKTAMDIFHVSEEDVTANMRRQAKAVNFGIVYGISDYGLAQNLNITRKEAAEFIERYFASFPGVKQYMDNIVQEAKQKGYVTTLLHRRRYLPDITSRNFNVRSFAERTAMNTPIQGSAADIIKKAMIDLSVRLREERLQARLLLQVHDELILEAPKEEIERLCRLVPEVMEQAVALRVPLKVDYHYGPTWYDAK* |
| R5_2_-mCh-FL-BST_LF_ | MGSSSKKSGSYSGSKGSKRRILSSKKSGSYSGSKGSKRRILNLVSKGEEDNMAIIKEFMRFKVHMEGSVNGHEFEIEGEGEGRPYEGTQTAKLKVTKGGPLPFAWDILSPQFMYGSKAYVKHPADIPDYLKLSFPEGFKWERVMNFEDGGVVTVTQDSSLQDGEFIYNVKLRGTNFPSDGPVMQKKTMGWEASSERMYPEDGALKGEIKQRLKLKDGGHYDAEVKTTYKAKKPVQLPGAYNVNIKLDITSHNEDYTIVEQYERAEGRHSTGGMDELYKGGGGSGGGGSGGGSNSTDEGEKPLAGMDFAIADSVTDEMLADKAALVVEVVGDNYHHAPIVGIALANERGRFFLRPETALADPKFLAWLGDETKKKTMFDSKRAAVALKWKGIELRGVVFDLLLAAYLLDPAQAAGDVAAVAKMHQYEAVRSDEAVYGKGAKRTVPDEPTLAEHLARKAAAIWALEEPLMDELRRNEQDRLLTELEQPLAGILANMEFTGVKVDTKRLEQMGAELTEQLQAVERRIYELAGQEFNINSPKQLGTVLFDKLQLPVLKKTKTGYSTSADVLEKLAPHHEIVEHILHYRQLGKLQSTYIEGLLKVVHPVTGKVHTMFNQALTQTGRLSSVEPNLQNIPIRLEEGRKIRQAFVPSEPDWLIFAADYSQIELRVLAHIAEDDNLIEAFRRGLDIHTKTAMDIFHVSEEDVTANMRRQAKAVNFGIVYGISDYGLAQNLNITRKEAAEFIERYFASFPGVKQYMDNIVQEAKQKGYVTTLLHRRRYLPDITSRNFNVRSFAERTAMNTPIQGSAADIIKKAMIDLSVRLREERLQARLLLQVHDELILEAPKEEIERLCRLVPEVMEQAVALRVPLKVDYHYGPTWYDAK* |

Table S3: Q5 high-fidelity PCR Temperature Profile

| **Step** | **Temperature** | **Time** | **Cycles** |
| --- | --- | --- | --- |
| Initial Denaturation | 98°C | 30s | 1 |
| Denaturation | 98°C | 10s | 30 |
| Annealing | 50°C | 30s |  |
| Extension | 72°C | 30s/kb |  |
| Final Extension | 72°C | 120s | 1 |

**
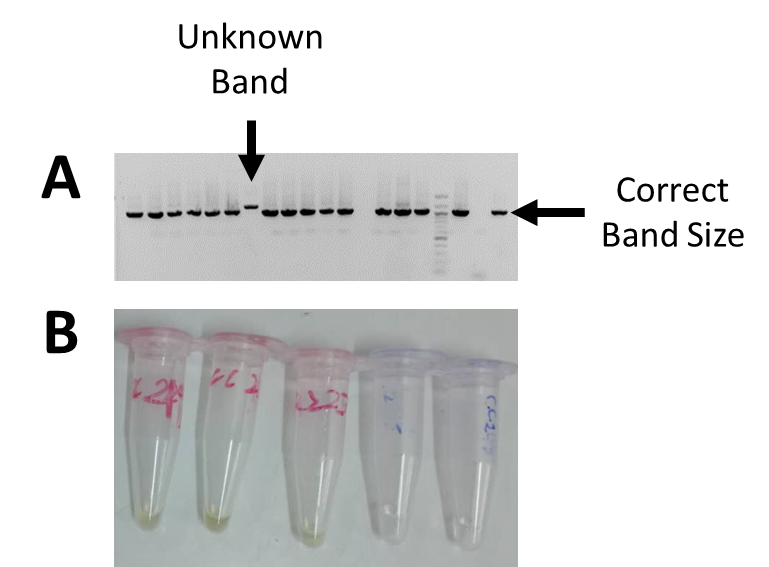
**

Figure S1: Picture of (A) PCR electrophoresis gel on a discounted sample due to the unknown amplification source and (B) the blood contaminated samples (left 3 eppendorfs) that inhibited Taq DNA polymerase.

Table S4: LAMP primer sequences designed by Lau et al. targeting the 18s rRNA gene of various infectious Plasmodium strains [1]. Forward outer primer – F3, reverse outer primer - B3, forward inner primer – FIP, reverse inner primer – BIP, forward loop primer – LPF, reverse loop primer – LPB.

Table S5: Additional primer sequences used for LAMP-based malaria diagnostics [2, 3]. Forward outer primer – F3, reverse outer primer - B3, forward inner primer – FIP, reverse inner primer – BIP, forward loop primer – LPF, reverse loop primer – LPB.

Table S6: Temperature profile for the BST activity-based qPCR assay.

| **Step** | **Temperature** | **Time** | **Cycles** |
| --- | --- | --- | --- |
| Initial Denaturation | 98°C | 30s | 1 |
| Denaturation | 98°C | 10s | 50 |
| Annealing | 53°C | 30s |  |
| Extension | 72°C | 30s |  |

Table S7: Temperature profile for PCR diagnostics of P. falciparum.

| **Step** | **Temperature** | **Time** | **Cycles** |
| --- | --- | --- | --- |
| Initial Denaturation | 95°C | 3s | 1 |
| Denaturation | 95°C | 15s | 50 |
| Annealing | 58°C | 60s |  |
| Extension | 68°C | 60s |  |
| Final Extension | 68°C | 5s | 1 |

Table S8: Temperature profile for qPCR diagnostics of P. falciparum.

| **Step** | **Temperature** | **Time** | **Cycles** |
| --- | --- | --- | --- |
| Initial Denaturation | 95°C | 120s | 1 |
| Denaturation | 95°C | 15s | 40 |
| Annealing | 60°C | 60s |  |
| Extension | 60°C | 60s |  |
| Final Extension | 60°C - 95°C |  | 1 |


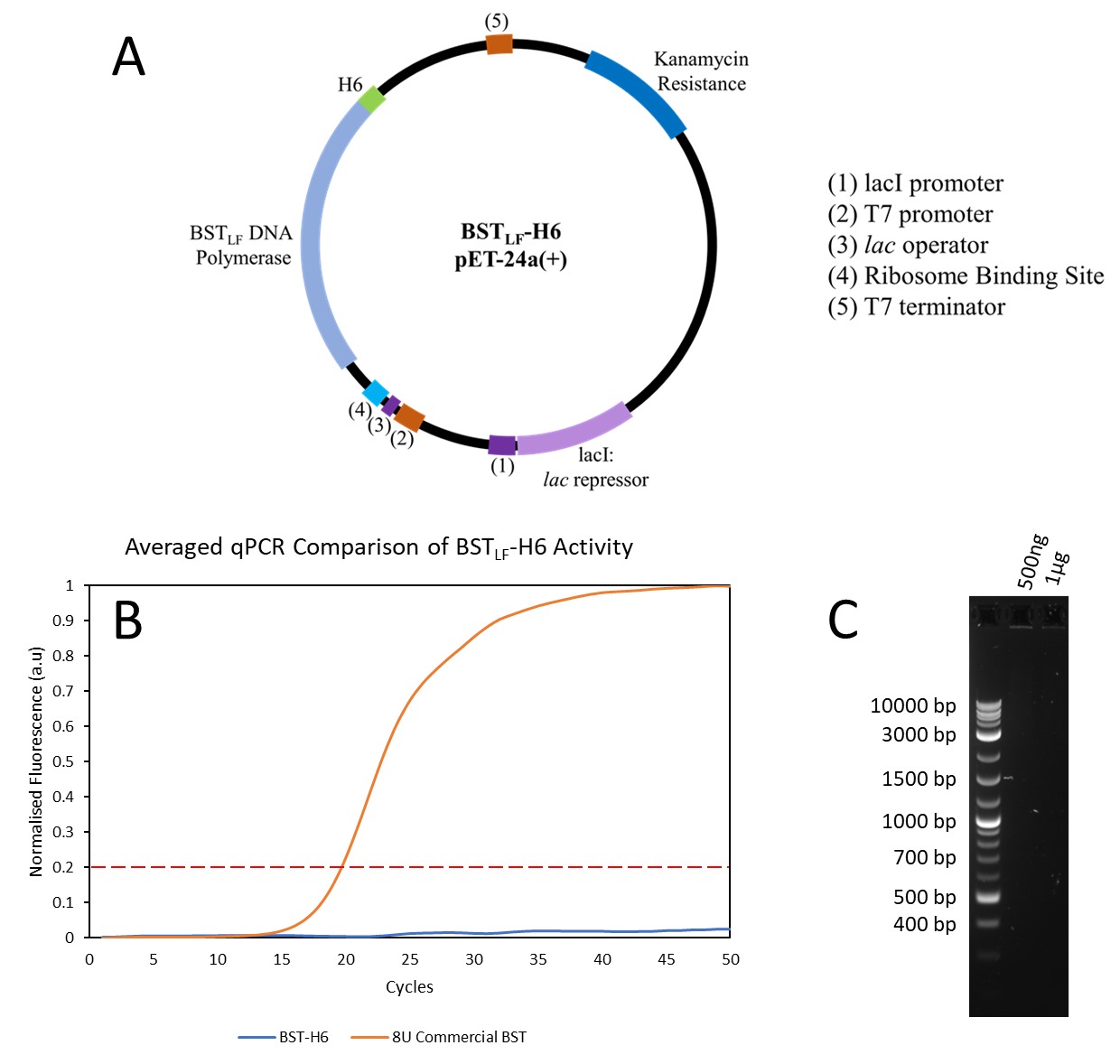


Figure S2: A: pET-24a(+) plasmid map illustrating the C-terminal modified BST_LF_. B: Averaged qPCR assay quantifying the amount of DNA produced by recombinant BST_LF_-H6 in comparison to a standard 8U of commercial BST_LF_. The initial strand invasion and displacement amplification assay targeted 100 copies of P. knowlesi using the F3 and B3 primers from the P.KNO-LAU primer set. C: 2% agarose gel electrophorese of BST_LF_-H6 LAMP assay targeting 10^6^ copies of P. knowlesi 18s rRNA using the P.KNO-LAU primer set.


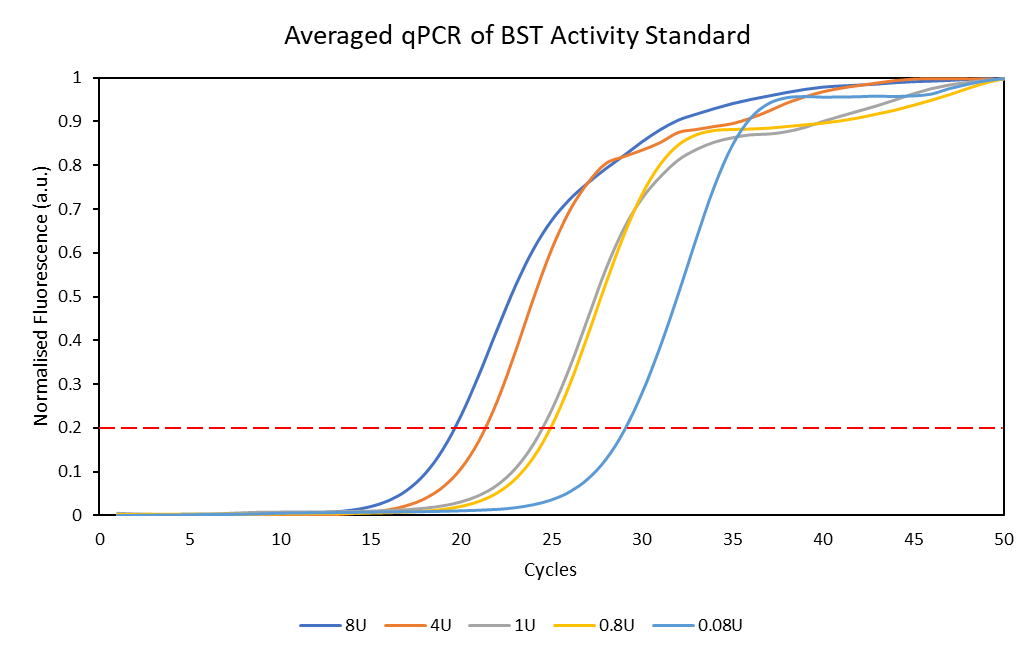


Figure S3: Averaged qPCR assay quantifying the amount of DNA produced by varying amounts of commercial BST_LF_. The resulting calibration line was equated to y=41616e^-0.441x^ with an R^2^ value of 0.64. 1U of BST_LF_ is defined as the amount of enzyme required to incorporate 10nmol of dNTP in 30 minutes at 65°C. The initial strand invasion and displacement amplification assay targeted 100 copies of P. knowlesi using the F3 and B3 primers from the P.KNO-LAU primer set.


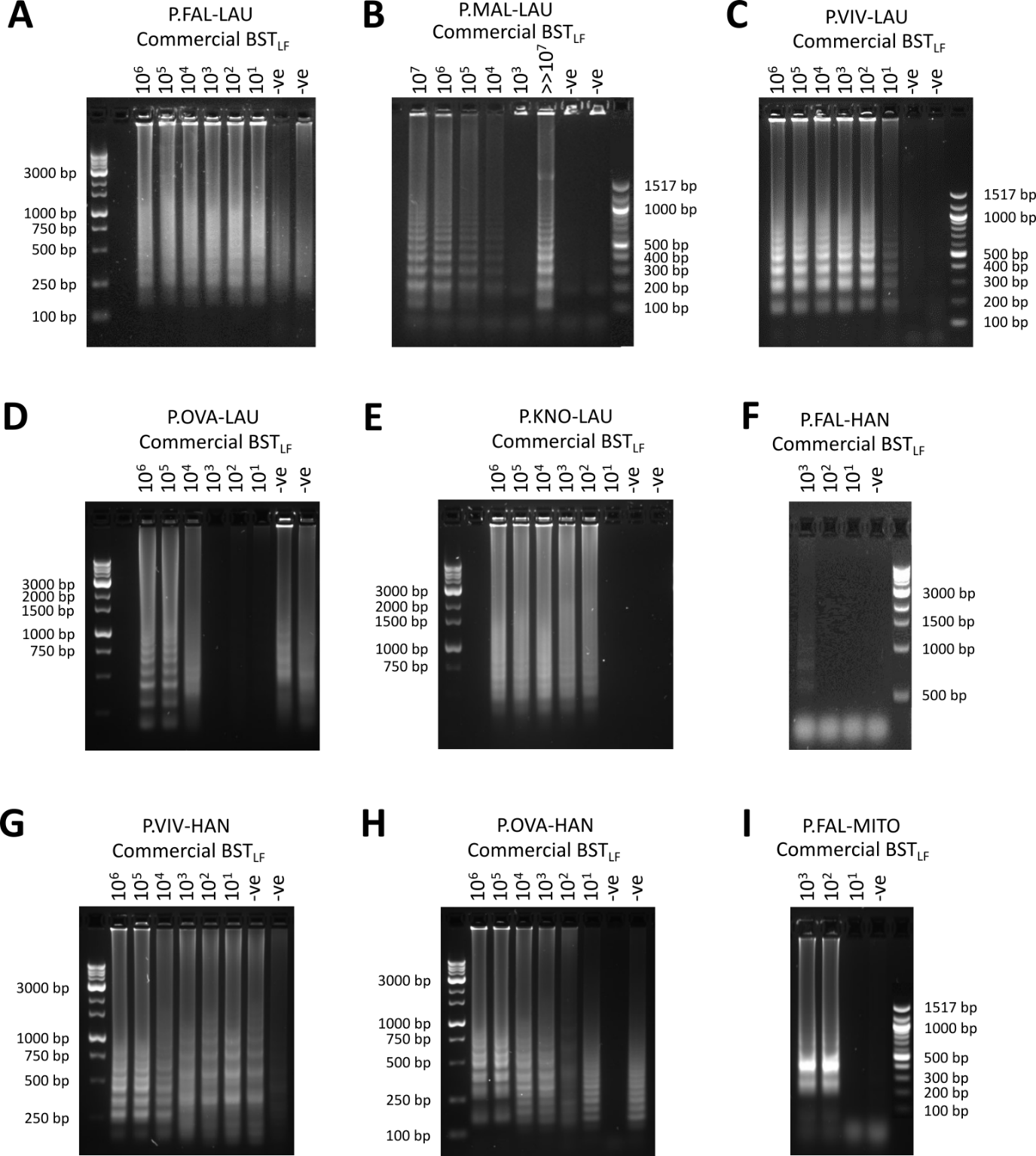


Figure S4: 2% agarose gel electrophoresis of 90-minute limit of detection LAMP assays for the malaria primer sets using 8U of commercial BST_LF_. Assays (A, D, G, H) all show false positives through a change in band pattern. Both assays (F, I) used genomic P. falciparum DNA instead of plasmid DNA. The number above each gel refers to the number of copies of plasmid or genomic DNA.


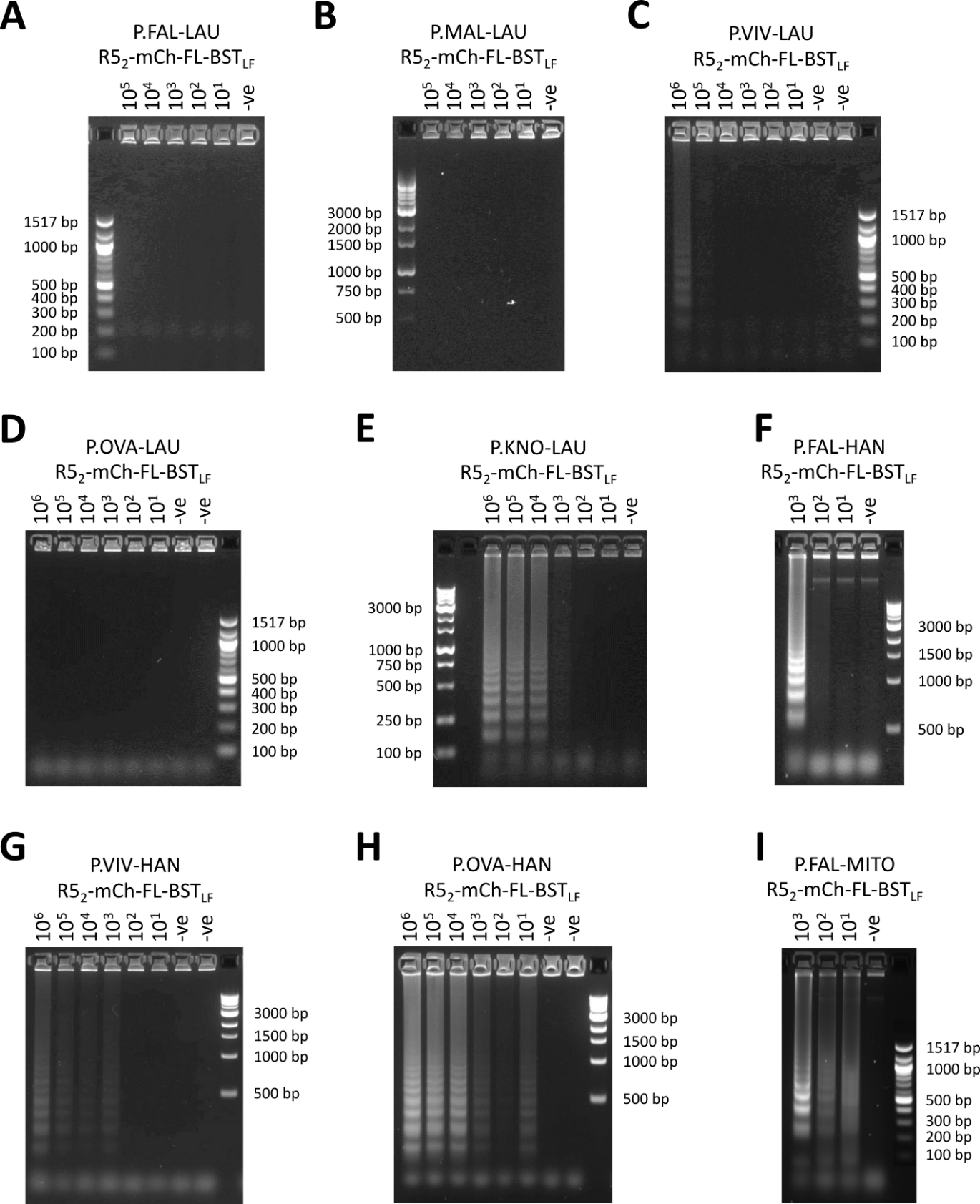


Figure S5: 2% agarose gel electrophoresis of 90-minute limit of detection LAMP assays for the malaria primer sets using 0.4mg of R5_2_-mCh-FL-BST_LF_. Both assays (F, I) used genomic P. falciparum DNA instead of plasmid DNA. The number above each gel refers to the number of copies of plasmid or genomic DNA.


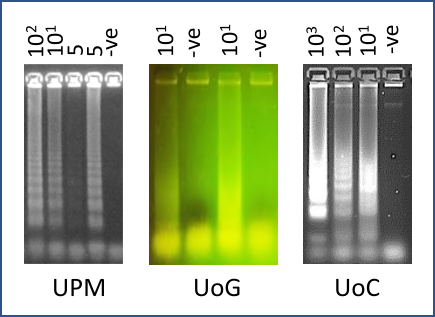


Figure S6: Gel electrophoresis of 90-minute LAMP reactions A: targeting P. falciparum mitochondrial Dd2 genomic DNA with the P.FAL-MITO primer set and using locally produced and silica immobilised R5_2_-mCh-FL-BST_LF_ in UPM, Selangor Malaysia, University of Ghana, Accra Ghana and University of Cambridge, Cambridge UK.

**References**

1. Lau Y-L, Lai M-Y, Fong M-Y, Jelip J, Mahmud R (2016) Loop-Mediated Isothermal Amplification Assay for Identification of Five Human Plasmodium Species in Malaysia. Am J Trop Med Hyg 94:336–339. https://doi.org/10.4269/ajtmh.15-0569

2. Han E-T, Watanabe R, Sattabongkot J, Khuntirat B, Sirichaisinthop J, Iriko H, Jin L, Takeo S, Tsuboi T (2007) Detection of Four Plasmodium Species by Genus- and Species-Specific Loop-Mediated Isothermal Amplification for Clinical Diagnosis. J Clin Microbiol 45:2521–2528. https://doi.org/10.1128/JCM.02117-06

3. Polley SD, Mori Y, Watson J, Perkins MD, González IJ, Notomi T, Chiodini PL, Sutherland CJ (2010) Mitochondrial DNA Targets Increase Sensitivity of Malaria Detection Using Loop-Mediated Isothermal Amplification. J Clin Microbiol 48:2866–2871. https://doi.org/10.1128/JCM.00355-10
